# Patient and Family Perspectives on Generative AI Tools in Rare Diseases: Exploratory Mixed Methods Online Survey

**DOI:** 10.2196/93720

**Published:** 2026-07-24

**Authors:** Charlotte Blease, James Jones, Catherine E Blease, Anna Kharko, Carolina Garcia Sanchez, Kenneth D Mandl

**Affiliations:** 1 Department of Women's and Children's Health, Uppsala University, MTC-huset, Dag Hammarskjölds väg 14B, 1 tr, Uppsala, Uppsala, 752 37, Sweden, 46 734697471; 2 Digital Psychiatry, Department of Psychiatry, Beth Israel Deaconess Medical Center, Boston, MA, United States; 3 Computational Health Informatics Program, Boston Children’s Hospital, Boston, MA, United States; 4 Centre for Primary Care and Health Services Research, University of Manchester, Manchester, England, United Kingdom; 5 Departments of Pediatrics and Biomedical Informatics, Harvard Medical School, Boston, MA, United States

**Keywords:** generative AI, general practice, primary care, large language models, education, training, online survey questionnaire, qualitative research

## Abstract

**Background:**

Generative artificial intelligence (GenAI) tools are widely accessible to the public, who are engaging with them for a wide range of health care applications. Existing research has focused predominantly on clinician-facing adoption. Far less is known about how patients and family members use GenAI tools, particularly in rare disease contexts, where diagnostic delay, limited specialist access, and unmet informational needs are common.

**Objective:**

This study aimed to examine the experiences and opinions of adult patients with rare diseases and parents or guardians of children with rare diseases regarding the use of GenAI tools.

**Methods:**

Between November 2025 and January 2026, we conducted an exploratory mixed methods web-based survey using convenience sampling through rare disease community organizations in the United States. The survey included closed-ended items assessing prior GenAI use, purposes of use, perceived influence on medical decisions and diagnoses, trust, concerns, communication with clinicians, and experiences of harm, alongside open-text questions capturing qualitative reflections. Descriptive statistics were used to summarize quantitative data. Inductive qualitative analysis was applied to the open-text responses.

**Results:**

A total of 115 respondents completed the survey. A majority of respondents were parents or guardians of a child with a rare disease (n=74, 64.3%), and the remaining respondents were patients with a rare disease (n=41, 35.7%). Slightly more than half of respondents (n=63, 54.8%) reported prior use of GenAI tools in the context of rare disease. Common purposes included exploring new treatments or clinical trials (n=53, 46.1%), interpreting medical tests or clinical notes (n=37, 32.2%), locating specialists or care centers (n=29, 25.2%), and suggesting possible diagnoses (n=28, 24.3%). Nearly one-third of respondents (n=37, 32%) reported some degree of influence of GenAI on their medical decisions. Nearly 10% (n=12) reported contributions of GenAI to a formal diagnosis. Concern about GenAI accuracy was widespread; 71 of 115 (61.8%) respondents reported moderate to extreme concern. Most respondents (n=90, 78.3%) had not discussed AI-generated information with a clinician. Few respondents (n=7, 6.1%) reported experiencing harm. Qualitative analysis identified 3 themes: (1) GenAI as a practical tool for augmenting patient and caregiver expertise and advocacy, (2) conditional trust and bounded use of GenAI with an emphasis on verification and human oversight, and (3) perceived risks, harms, and structural concerns, including inaccuracies, genetic misinterpretation, and privacy and commercialization issues.

**Conclusions:**

In this exploratory study, patients and families affected by rare diseases were actively experimenting with GenAI tools to support information seeking, preparation, and advocacy while simultaneously expressing substantial caution and concern about the reliability, safety, and appropriate boundaries of use. Our findings contrast sharply with clinician concerns that patients lack the capacity to use GenAI tools judiciously. Notwithstanding, the sample was skewed toward highly educated participants. Future research should prioritize more representative samples to better capture the range of patient and caregiver experiences with GenAI in rare disease care.

## Introduction

### Background

Rare diseases collectively affect an estimated 10% of the global population, corresponding to approximately 500 million people worldwide [1]. Although individually uncommon, rare diseases constitute a large and heterogeneous group, with approximately 7000 distinct conditions currently described [2]. However, the distribution of cases is highly skewed: roughly 350 rare diseases account for around 80% of diagnoses, while thousands of additional conditions are classified as ultrarare [2]. Definitions of rarity vary internationally, with different taxonomic thresholds, including fewer than 1 in 2000 individuals in the European Union, fewer than 200,000 people in the United States, and fewer than 50,000 people in Japan [1,3]. In addition, genetic rare diseases differ across geographic regions, making the challenge of clinician diagnoses even greater in many cases. Together, these figures illustrate that rare diseases are neither exceptional nor marginal in aggregate, yet they remain structurally underrecognized within health care systems.

People with rare diseases frequently experience prolonged and complex diagnostic pathways. Substantial delays between symptom onset and correct diagnosis are common. A study in Australia found that approximately 30% of individuals waited between 5 and 30 years for a definitive diagnosis [4]. In the United States, another study reported that around 50% of patients and caregivers attribute diagnostic delays to limited clinician awareness of their condition [5]. These challenges occur within a clinical environment characterized by the rapid expansion of biomedical knowledge. In addition to thousands of existing rare diseases, approximately 250 new rare diseases are described each year, accompanied by the continual refinement of gene-disease associations and molecular subtypes [2]. Under these conditions, it has been argued that maintaining up-to-date familiarity across rare disease domains is not realistically achievable for individual clinicians [6].

Even when an accurate diagnosis is achieved, therapeutic options are often limited. Fewer than 10% of rare diseases currently have an available disease-modifying treatment, and approximately 95% lack a US Food and Drug Administration–approved therapy [2]. Evidence generation is constrained by small patient populations, fragmented research efforts, and high rates of trial noncompletion or nonpublication [7,8]. As a result, patients and families commonly engage in sustained self-directed information seeking, including monitoring emerging research, identifying potential clinical trials, and synthesizing highly technical biomedical information to support care decisions [9].

In parallel, rare disease care has long challenged conventional assumptions about where medical expertise resides. In part, because of knowledge deficits among clinicians and diagnostic delays, patient-researchers argue that people living with chronic and rare conditions often develop deep, experience-based expertise and may actively contribute to research, innovation, and care improvement rather than merely receiving it [9,10]. However, health care systems frequently struggle to recognize patient and caregiver knowledge. Philosophers describe this problem as one of “epistemic injustice”—a “wrong done to someone in their capacity as a knower,” in which patients’ accounts tend to be granted less credibility than professional perspectives [11,12]. In clinical settings, this can manifest as “testimonial injustice” (patients not believed) or “hermeneutical injustice” (patients’ experiences not fitting existing medical categories), phenomena closely related to what patients often describe as medical gaslighting [11,13]. Set against these concerns, the rapid public availability of large language model (LLM)–powered chatbots has burgeoned when it comes to health-related searches [14,15]. The use of consumer generative artificial intelligence (GenAI) tools, such as ChatGPT, Gemini, and DeepSeek, and a range of other models has created new possibilities for processing health information. Public access to consumer GenAI tools is a recent phenomenon. ChatGPT, the first widely available LLM chatbot, was released by OpenAI in November 2022. This prompted the rapid release of competing tools, including Google’s Gemini (2023) and Anthropic’s Claude (2023), among others. GenAI tools are, therefore, readily available but are susceptible to producing inaccurate or fabricated outputs and may encode or amplify existing biases, including those related to race, gender, and disability, with potential consequences for equity in health care [16]. In addition, the widespread availability of consumer-facing GenAI tools, coupled with their highly interactive conversational interfaces, raises concerns about privacy, data security, and the handling of sensitive personal health information [17,18].

To date, empirical research on GenAI in health care has primarily examined clinician adoption of both consumer and medical-grade applications for a range of clinical tasks, including documentation support, clinical reasoning, and treatment recommendations [19]. In contrast, far less is known about how patients and family members use GenAI tools [19-22]. This gap is particularly consequential for rare disease communities, where informational needs are persistent and clinical expertise is unevenly distributed [20]. However, one US survey found that households with rare diseases were twice as likely to use GenAI tools compared with other households [23].

Moreover, even before GenAI, people affected by rare diseases were established users of online resources—both to manage the chronic uncertainty of conditions with life-limiting potential, such as cancer predisposition syndromes and degenerative disorders [24-26], and to connect with peers and advocacy organizations for social support [27-30]. Consumer GenAI tools thus enter an information ecosystem in which these communities are already highly engaged online.

Notably, parents in rare disease communities have a documented history of being early adopters of novel and at times controversial technologies [31], suggesting that engagement with emerging GenAI tools is consistent with longstanding patterns of technology uptake in this population. This study takes epistemic injustice as its theoretical framework, asking how patient and caregiver GenAI use intersects with the credibility deficits and interpretive gaps common among patients with rare diseases, many of whom wait decades to be diagnosed [4,5].

### Objective

Against the theoretical framework of epistemic injustice and the research gaps related to patient use of GenAI, the objective of this mixed methods study was to examine the experiences and opinions of adult patients with rare diseases and parents or guardians of children with rare diseases regarding the use of GenAI tools. Specifically, we asked the following questions: *How do patients and family members in rare disease communities use GenAI tools? What purposes do these tools serve? What benefits and concerns are perceived? To what extent do participants report that AI-generated information influences health care decisions or interactions with clinicians?* We addressed these questions using a convenience sample recruited through rare disease networks and community organizations in the United States, combining quantitative survey data with open-text qualitative responses.

## Methods

### Study Design and Ethics

This study used an exploratory mixed methods cross-sectional survey design to examine the experiences and opinions of patients with rare diseases and parents or guardians of children with rare diseases regarding the use of GenAI tools in the context of rare disease care. A mixed methods approach was selected to enable descriptive quantification of patterns of use alongside qualitative exploration of concerns and contextual experiences. Given the limited existing patient-centered evidence in this area, an exploratory design was considered appropriate.

### Ethical Considerations

The study received ethics approval and was deemed exempt from the human participants research requirements of 45 CFR 46 by the Boston Children’s Hospital Institutional Review Board (approval ID IRB-P00051505). Electronic informed consent was obtained from all participants prior to survey initiation. Participation was voluntary, and respondents could withdraw at any time before submitting the survey. In addition, no compensation was provided for completing the survey.

### Survey Development

An original survey instrument was developed by the study team to address the study objectives. Survey content was informed by prior literature on patient information-seeking behaviors, emerging scholarship on GenAI in health care, and consultation with rare disease community stakeholders [23,32,33]. We distinguish here between our patient partner (CEB), who contributed to survey co-design, and the survey respondents (N=115), who participated anonymously. Specifically, our patient partner (CEB) with lived experience of a rare disease contributed to item development, wording, and assessment of relevance. Draft versions of the survey were reviewed by contacts within participating rare disease networks to assess clarity, accessibility, and face validity, and the instrument was timed to ensure feasibility of completion within approximately 5 minutes.

The final survey comprised 19 primary items, including multiple-choice, Likert-type, and checkbox questions, together with 4 standalone open-text free-response questions (items 10a, 16a, 17a, and 18). Open-text questions did not accompany every closed-ended item; rather, item 10a invited respondents to specify other purposes of GenAI use, whereas items 16a, 17a, and 18 invited extended reflection on experiences of harm, factors influencing cautious or nonuse, and final reflections on rare disease care and the potential role of GenAI tools, respectively. The 3 additional reflection questions were as follows:

16a. Please briefly describe the harm or negative outcomes you experienced from generative AI advice or information.
17a. Please describe other factors that have influenced your decision not to use generative AI tools or to use them more cautiously.
18a. Is there anything else you would like to share about your experiences with rare disease care, or your perspectives on the potential role of generative AI in this area?

All questions were optional. Because the study did not aim to examine identity-based differences and sought to reduce the collection of potentially sensitive personal data, gender was not included among the demographic items. The full survey instrument and informed consent materials are provided in Multimedia Appendix 1. The study adhered to the CHERRIES (Checklist for Reporting Results of Internet E-Surveys) guidelines [34] (Checklist 1).

### Participants and Recruitment

Participants were eligible if they were aged 18 years or older and self-identified as either a patient with a rare disease or a parent or guardian of a child with a rare disease. No additional exclusion criteria were applied. Recruitment used convenience sampling through the established rare disease networks and community organizations COMBINEDBrain [35] and EveryLife Foundation [36]. Based in the United States, both are umbrella networks of rare genetic disease communities with validated patient and family member contacts. Members of these organizations were invited by email to distribute an anonymous survey link through their email lists and closed-network newsletters, and with 2 reminder emails requested during the recruitment period. To intentionally reduce the risks of bot contamination, duplicate participation, and unverifiable eligibility, the survey team and contacts were requested not to share the survey link on any open social media platforms.

### Data Collection

The survey was hosted on REDCap (Vanderbilt University), which is a secure web-based data capture platform. Data collection occurred between November 1, 2025, and January 10, 2026. No personally identifiable information, including names, email addresses, or IP addresses, was collected, and responses were anonymous. In addition to fixed-response items, as noted previously, participants could provide open-text responses describing other purposes for GenAI use, experiences of harm or negative outcomes, additional factors influencing cautious use or nonuse, and any further reflections on rare disease care or the potential role of GenAI tools. To reduce the risk of reidentification, particularly given the rarity of some conditions and the potential for triangulation with demographic information, we did not share row-level raw data. Instead, we provided the full aggregated and tabulated responses in Multimedia Appendix 2.

### Data Analysis

Quantitative data were analyzed using descriptive statistics, including frequencies and percentages, to summarize respondent characteristics, prior use of GenAI, purposes of use, perceived influence on medical decisions, trustworthiness, concerns, and communication with health care professionals. Given the exploratory nature of the study and the limited expected sample size, no inferential statistical analyses were planned.

Qualitative thematic analysis was restricted to the 3 reflective free-response items (items 16a, 17a, and 18). Responses to item 10a (other purposes of use) consisted of brief specifications that supplemented the quantitative data on purposes of use and were not subjected to thematic analysis. Open-text responses were analyzed using a qualitative descriptive approach with inductive thematic coding, allowing patterns and concepts to be identified directly from the data rather than from pre-existing frameworks [37,38]. However, given the concise nature of many responses and the frequent use of brief phrases or sentence fragments, the data were not suited for in-depth interpretive thematic analysis [39]. Prior to analysis, responses indicating no substantive content (eg, “none,” “not applicable,” or “no comment”) were removed. The remaining responses were imported into an Excel file for data management and coding.

Two members of the research team (CB and CEB) independently reviewed the responses multiple times to develop familiarity with the dataset. CB is a philosopher, ethicist, and informaticist, and CEB is her twin sister, a government worker, who lives with a rare illness. An inductive coding strategy was then applied, whereby short descriptive codes were assigned to capture the central idea expressed in each response. Responses that conveyed more than one idea were assigned multiple codes. Coding was iterative, with ongoing comparison of codes to identify areas of convergence and divergence. CB and CEB met to discuss coding decisions, resolve only minor and trivial discrepancies, and refine the coding framework. Given the short and often fragmentary nature of the open-text responses, formal intercoder reliability statistics and thematic saturation were not appropriate because these techniques presuppose richer narrative data than this instrument was designed to elicit. Final codes were subsequently grouped into higher-order categories based on conceptual similarity, generating a structured summary of participant perspectives. Again, this approach is entirely consistent with the limitations of the dataset [39] and has been undertaken in previous analyses by the study team [40]. Owing to the online survey format and the limited responses, we were unable to establish thematic saturation.

## Results

### Sample Characteristics

A total of 115 respondents completed the survey. The majority of respondents (n=74, 64.3%) identified as parents or guardians of a child with a rare disease, whereas the remainder (n=41, 35.7%) identified as patients with a rare disease (Table 1). Participants were predominantly adults, with 61.7% (n=71) aged between 35 and 54 years. Educational attainment was high, with 75.7% (n=87) reporting a university or postgraduate degree. Most respondents resided in the United States (n=98, 85.2%). A breakdown of the results by respondent group is further presented in Multimedia Appendix 3.

**Table 1.** Respondent characteristics (N=115).

Characteristic	Total, n (%)
Age (y)	
18-24	2 (1.7)
25-34	18 (15.7)
35-44	35 (30.4)
45-54	36 (31.3)
55-64	16 (13.9)
65 or older	8 (7)
Highest attained education	
Secondary school	1 (0.9)
College or vocational training	27 (23.5)
University degree	41 (35.7)
Postgraduate degree	46 (40)
Country	
United States	98 (85.2)
United Kingdom	4 (3.5)
Australia	3 (2.6)
Canada	2 (1.7)
Colombia	1 (0.9)
Hungary	1 (0.9)
Israel	1 (0.9)
Lithuania	1 (0.9)
Pakistan	1 (0.9)
Spain	1 (0.9)
Turkey	1 (0.9)
Missing	1 (0.9)

### Diagnostic Journey

Respondents reported consulting a mean of 7.3 (SD 12.0) health care professionals prior to receiving a correct diagnosis (Table 2). Diagnostic timelines were frequently prolonged, with 43 of 115 (37.4%) reporting a wait of 3 years or longer, including 16 of 115 (13.9%) who reported delays exceeding 10 years. Among the participants, 22 of 115 (19.1%) reported receiving an official rare disease diagnosis since 2023.

**Table 2.** Diagnoses and time to diagnosis (N=115).

Characteristic	Total, n (%)
Diagnosis^a^	
Tuberous sclerosis complex	31 (26.1)
Stiff person syndrome	13 (11.3)
Leber hereditary optic neuropathy	9 (7.8)
SYNGAP1-related disorder	6 (5.2)
Angelman syndrome	5 (4.3)
Okur-Chung syndrome	2 (1.7)
DYNC1H1-related disorder	2 (1.7)
Hao-Fountain syndrome	2 (1.7)
SLC6A1-related disorder	2 (1.7)
Epilepsy (Grand Mal)	2 (1.7)
Other single-entry diagnoses	41 (35.7)
Missing	1 (0.9)
Time to diagnosis (y)	
Less than a year	45 (39.1)
1-2	26 (22.6)
3-5	15 (13)
6-10	12 (10.4)
More than 10 years	16 (13.9)
Missing	1 (0.9)

a Multiple diagnoses could be reported; the total count will exceed the number of participants. All reported diagnoses can be found in Multimedia Appendix 2.

### Use of GenAI Tools

Slightly more than half of the respondents (63/115, 54.8%) reported some prior use of GenAI tools for rare disease–related purposes, whereas 52 of 115 (45.2%) respondents reported never using such tools (Table 3). Among users, the most commonly reported purposes were exploring new treatments or clinical trials (53/63, 84.1%) and interpreting medical tests or clinical notes (37/63, 58.7%). One-third of respondents (37/115, 32.2%) reported additional uses, including summarizing scientific publications and supporting insurance appeals or advocacy.

**Table 3.** Use of generative artificial intelligence (GenAI) tools (N=115).

Characteristic	Total, n (%)
Prior use of GenAI tools	
Never	52 (45.2)
Occasionally	21 (18.3)
Once or twice	17 (14.8)
Regularly	25 (21.7)
Reasons for using GenAI tools^a^	
Exploring new treatments or clinical trials	53 (46.1)
Interpreting medical tests or notes	37 (32.2)
Finding specialists or care centers	29 (25.2)
Suggesting possible diagnoses	28 (24.3)
Emotional or social support or advice	18 (15.7)
Preparing questions for health care visits	17 (14.8)
Other	37 (32.2)

a Multiple reasons could be reported; the total count will exceed the number of participants.

### Influence of GenAI on Medical Decisions and Diagnosis

As shown in Table 4, most respondents reported minimal or no influence of GenAI tools on medical decisions (77/115, 67.0%). However, nearly one-third of respondents (37/115, 32.2%) reported that GenAI had influenced their medical decisions to some degree, including 10 (8.7%) respondents who reported a strong influence. Direct contributions of GenAI to formal diagnoses were uncommon (11/115, 9.6% reporting any contribution), and most respondents reported that GenAI had not shortened their diagnostic journey (93/115, 80.9%).

**Table 4.** Influence of generative artificial intelligence tools on medical decisions and diagnoses (N=115).

Characteristic	Total, n (%)
Influence on medical decisions	
Minimal or no influence	77 (67.0)
Somewhat	27 (23.5)
Strongly	10 (8.7)
Missing	1 (0.9)
Contribution to formal diagnosis	
Did not help	90 (78.3)
Somewhat helpful	7 (6.1)
Critical contribution	4 (3.5)
Unsure	13 (11.3)
Missing	1 (0.9)
Shortened diagnostic journey	
Not noticeably	93 (80.9)
Moderately	8 (7.0)
Significantly	3 (2.6)
Unsure	9 (7.8)
Missing	2 (1.7)

### Trust, Concerns, and Communication With Clinicians

Perceived trustworthiness of AI-generated health information was mixed: of 115 respondents, 45 (39.1%) respondents rated it as less trustworthy than traditional sources, while 33 (28.7%) rated it as equally trustworthy (Table 5). Concern about accuracy was widespread, with 71 (61.7%) respondents reporting moderate, very high, or extreme concern. Notably, most respondents (n=90, 78.3%) had not discussed AI-generated information with a health care professional.

**Table 5.** Trust, concerns, and communication regarding generative AI tools (N=115).

Characteristic	Total, n (%)
Perceived trustworthiness of AI-generated health information compared to traditional sources	
Less trustworthy	45 (39.1)
Equally trustworthy	33 (28.7)
More trustworthy	7 (6.1)
Unsure	30 (26.1)
Concerns with accuracy of AI-generated medical information	
Extremely concerned	18 (15.7)
Very concerned	22 (19.1)
Moderately concerned	31 (27.0)
Slightly concerned	31 (27.0)
Not concerned	12 (10.4)
Missing	1 (0.9)
Discussions of AI-generated information with health care provider	
Not discussed	90 (78.3)
Health care provider was skeptical	9 (7.8)
Health care provider was supportive	14 (12.2)
Missing	1 (0.9)

### Experience of Harm

Few respondents reported experiencing harm or negative outcomes related to GenAI use (7/115, 6.1%; Table 6). When considering factors that dissuade respondents from using GenAI tools, most cited uncertainty about accuracy (75/115, 65.2%), followed by privacy concerns (36/115, 31.3%) and lack of trust (35/115, 30.4%).

**Table 6.** Experience of harm or negative outcomes related to generative artificial intelligence (GenAI) tools (N=115).

Characteristic	Total, n (%)
Experience of harm or negative outcomes from GenAI advice or information	
Yes	7 (6.1)
No	105 (91.3)
Missing	3 (2.6)
Factors that influence the decision not to use GenAI tools or to use them more cautiously	
Uncertainty about accuracy	75 (65.2)
Privacy concerns	36 (31.3)
Lack of trust	35 (30.4)
Technological comfort or skill	11 (9.6)
Lack of awareness or access	10 (8.7)
None, use without hesitation	21 (18.3)
Other	7 (6.1)

### Qualitative Component

#### Overview of Qualitative Findings

**Figure 1.**
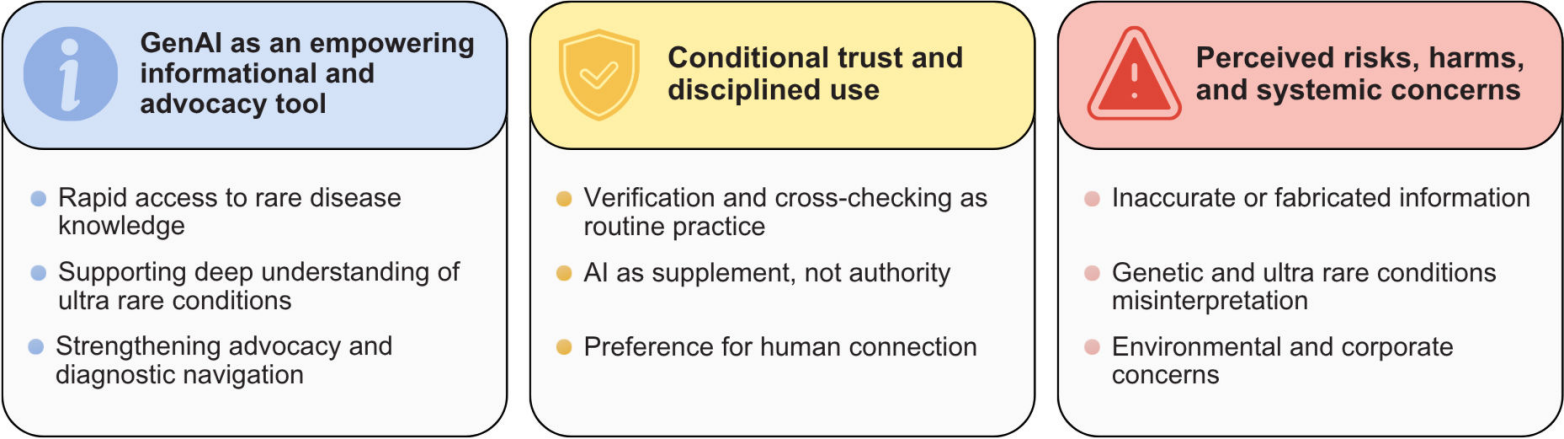
Themes and subthemes. GenAI: generative artificial intelligence.

A total of 66 respondents provided substantive open-text comments across 3 survey items addressing GenAI tools in relation to experiences of harm (item 16a), additional factors influencing cautious or nonuse of GenAI tools (item 17a), and final reflections on rare disease care and the potential role of GenAI tools (item 18). As a result of using an inductive thematic coding, 3 overarching themes and associated subthemes were identified (Figure 1), which are presented below with illustrative quotations. Parenthetical numbers indicate individual participant identifiers; also indicated are age group and whether respondents reported being a patient with a rare disease or a parent or guardian of a child with a rare disease.

#### Theme 1: GenAI as an Empowering Informational and Advocacy Tool

##### Overview

Participants frequently described GenAI as a practical resource for navigating complex rare disease information, supporting learning, and strengthening medical self-advocacy or caregiver-advocacy. Crucially, these accounts position GenAI as a response to a structural deficit—scarce expertise and limited clinical time—rather than as a substitute for professional judgment.

##### Subtheme 1.1: Rapid Access to Rare Disease Knowledge

A recurring emphasis in participants’ accounts was the speed and accessibility of information that GenAI provided—particularly in contexts where clinical time is limited and rare disease expertise is scarce:

*AI helps to find extra information really fast*.

[#41, 45-54 years, Parent/Guardian]

*It’s been helpful for asking a million questions the doctors don’t have time for*.

[#92, 35-44 years, Parent/Guardian]

The second account is especially revealing: it locates GenAI’s value not in replacing the clinician but in absorbing the questions that the clinical encounter cannot accommodate. Against our finding that respondents consulted a mean of 7.3 (SD 12.0) clinicians before diagnosis, this points to GenAI filling the interstitial spaces of fragmented care pathways.

Several respondents underscored the value of rapid access in contrast with their own diagnostic histories, expressing regret that these tools had not been available to them earlier:

*My son was diagnosed before AI in 2018. But I would have used AI if it were available back then to try and find the answers.*

[#4, 35-44 years, Parent/Guardian]

*At the time of my daughter’s diagnosis, this was not available. But I am also a rare disease patient,...and do see the value when looking for quick information.*

[#12, 35-44 years, Parent/Guardian]

##### Subtheme 1.2: Supporting a Deep Understanding of Ultrarare Conditions

Beyond rapid retrieval, several respondents described GenAI as enabling qualitatively deeper engagement with genetic and mechanistic detail—knowledge ordinarily gated behind specialist channels:

*Generative AI has taught me about the gene, the related pathway, other genes/disorders associated with the pathway and has helped me identify a gap in care*...

[#7, 45-54 years, Parent/Guardian]

*Generative AI has a great potential to democratize scientific knowledge to parents and caregivers (NotebookLM for example does a great job of breaking down dense research papers)*...

[#37, 35-44 years, Parent/Guardian]

These accounts suggest that GenAI functioned less as a search engine and more as a translation layer between specialist literature and lay expertise. The reference to identifying “a gap in care” is notable: the participant is not merely consuming information but is using it to audit the adequacy of care, an act of epistemic agency that the framework of epistemic injustice predicts patients are often denied the standing to perform.

##### Subtheme 1.3: Strengthening Medical Self-Advocacy and Caregiver-Advocacy and Diagnostic Navigation

A few respondents reported that GenAI had directly supported diagnostic reasoning or advocacy:

*I use AI almost every day and in virtually every facet of my life, including for rare disease care management, research, and advocacy activities.*

[#99, 35-44 years, Parent/Guardian]

*I entered all my past medical labs and notes from docs. ChatGPT was able to pinpoint my disease and was correct when I finally found a specialist that knew my disease.*

[#63, 45-54 years, Patient]

*AI wasn't around when we were seeking a diagnosis for my son…I would've been able to push harder against the doctors who were dismissing his symptoms*.

[#40, 35-44 years, Parent/Guardian]

The final quotation is the clearest articulation of GenAI as epistemic scaffolding—resources that can help patients to articulate and validate observations that might otherwise be dismissed: the participant frames it not as a source of answers but as a source of *standing*—the confidence and evidentiary footing to contest clinical dismissal. This directly engages with the testimonial dimension of epistemic injustice, where patients’ accounts are given diminished credibility by clinicians [12,41,42]. Here, GenAI is imagined as a counterweight to that asymmetry, although the same accounts also caution that such confidence must be calibrated against the reliability concerns documented in Theme 3.

### Theme 2: Conditional Trust and Disciplined Use

#### Overview

If Theme 1 describes the reach of GenAI use, Theme 2 describes its limits, and these limits were self-imposed and explicit. Participants did not describe trust as a binary but as a conditional, effortful stance that had to be earned through verification and was bounded by clear rules about where the GenAI tool’s authority ended.

#### Subtheme 2.1: Verification and Cross-Checking as Routine Practice

Respondents described verification not as an occasional safeguard but as standard operating procedure:

*Important to include sources for AI responses and then check those sources. I also use two or more AI tools as cross reference and frequently ask for clarifications or restatements*.

[#38, 65 years or older, Parent/Guardian]

*I always ask for links or references to verify what it is telling me is accurate*.

[#3, 35-44 years, Parent/Guardian]

*Ask for sources and cross reference and challenge.*

[#18, 65 years or older, Parent/Guardian]

These are not the practices of uncritical users. The recurrence of triangulation, source-checking, and adversarial questioning (“challenge”) indicates a working approach among participants whereby GenAI output is treated as a hypothesis to be tested rather than a conclusion to be accepted. This directly counters deficit framings that assume lay users absorb AI output passively. It is notable, however, that the verification described here may operate within a closed informational loop: participants reported triangulating across multiple AI systems and sought online sources and references, rather than cross-checking GenAI output against real-world sources such as clinicians. Whether they raised informational queries in a more clandestine way with clinicians, however, is a possibility.

#### Subtheme 2.2: AI as Supplement, Not Authority

Participants drew a firm line between informational support and clinical authority:

*I would not trust my child’s healthcare to AI and would not trust a provider who used AI as anything more than a source of an idea to consider*.

[#17, 45-54 years, Parent/Guardian]

*I will never trust generative AI as a source when so many wonderful research summaries are available that are written by human scientists*.

[#16, 55-64 years, Parent/Guardian]

The first quotation is striking for extending the boundary to clinicians themselves—the participant polices not only their own reliance on GenAI but their providers’ as well. This suggests that, for at least some participants, the appropriate role of GenAI is a settled normative question, not an open one.

#### Subtheme 2.3: Preference for Human Connection

Human interaction remained central to several participants’ conceptions of care:

*I feel the human interaction is the best way to receive care. Human touch is key to connection. It’s genuine, not artificial.*

[#88, 45-54 years, Patient]

Here, the limit on GenAI is about what it cannot offer. This participant values human care for its own sake. This suggests that, for some participants, GenAI use and the clinical relationship need not be in competition.

### Theme 3: Perceived Risks, Harms, and Systemic Concerns

#### Overview

The caution evident in Theme 2 was grounded in concrete experience, as further elaborated in Theme 3. Participants’ concerns ranged from specific encounters with errors to structural critiques of the technology’s social costs.

#### Subtheme 3.1: Inaccurate or Fabricated Information

Several respondents reported direct encounters with errors, including hallucinated references and incorrect clinical content:

*It has generated nonexistent references mixing authors and titles and journals, etc*.

[#36, 45-54 years, Parent/Guardian]

*Provided incorrect information about therapy option*.

[#5, 35-44 years, Parent/Guardian]

*AI seems to be giving me the information that is popular, but not accurate*.

[#72, 55-64 years, Patient]

The final observation—that GenAI may prioritize prevalence over precision—is particularly pertinent in rare disease contexts, where conditions are, by definition, underrepresented in training data.

#### Subtheme 3.2: Genetic and Ultrarare Misinterpretations

A subset of participants with genetic expertise described technically sophisticated errors that nonspecialists could not easily detect:

*It has given incorrect information for example the number of exons in the MED13L gene or it has read a genetic report that was a frameshift and deemed it a nonsense*.

[#10, 35-44 years, Parent/Guardian]

*When using it to research ultrarare diseases and specifically genetic variants, I have come across several inaccuracies*.

[#58, 35-44 years, Parent/Guardian]

This subtheme reveals a basic problem: GenAI may be least reliable for exactly the rare conditions where patients most need accurate information. Moreover, the errors are hard to detect. These participants identified them only because they had unusual technical expertise; most users might not have done so. This is what makes the verification practices in Theme 2 necessary, and it tempers the more optimistic picture in Theme 1.

#### Subtheme 3.3: Environmental and Corporate Concerns

A minority of respondents raised broader ethical objections:

*The environmental impact of the data centers. Making billionaires even richer*.

[#63, 45-54 years, Patient]

*They are destroying the environment and contributing to environmental racism*.

[#113, 35-44 years, Patient]

Although raised by few respondents, these accounts indicate that GenAI was, for some, evaluated against a wider ethical horizon than individual accuracy. That such critiques surface in a rare disease sample is itself notable: communities already attuned to systemic inequity in health care appear to extend that scrutiny to the political economy of the tools they nonetheless use—a further marker of the critical, nonnaive engagement that characterizes the dataset.

## Discussion

### Principal Findings

Currently, there is very little research on patient use of GenAI tools, particularly among people affected by rare diseases, a historically marginalized population that must often navigate care amid limited clinician expertise. In this convenience sample, mixed methods survey (N=115), just over half of the respondents (n=63, 54.8%) reported using GenAI for rare disease-related tasks, most commonly exploring treatments or trials (n=53, 46.1%), interpreting medical tests or notes (n=37, 32.2%), identifying specialists (n=29, 25.2%), and considering possible diagnoses (n=28, 24.3%). Qualitative responses indicated these activities functioned as extensions of caregiving and medical self-advocacy—helping participants understand complex genetic information and prepare questions—rather than as attempts to replace clinicians.

Participants used GenAI cautiously and strategically. Most reported minimal or no influence on medical decisions (n=77, 67.0%), formal diagnostic contributions were uncommon (n=11, 9.6%), and concern about accuracy was widespread (n=71, 61.8%). Few had discussed AI outputs with clinicians (n=90, 78.3%), and harms were rarely reported (n=7, 6.1%). Against a backdrop of prolonged diagnostic effort (clinicians consulted: mean 7.3, SD 12.0; n=43, 37.4% waiting ≥3 y), GenAI primarily served as a cognitive support for navigating fragmented care and potentially constrained access to expertise—enabling preparation, verification, and self-advocacy in medicine while maintaining reliance on human clinical oversight.

A large probability-based US survey conducted earlier in 2025 found that people in households with members having a rare disease were disproportionately early adopters of digital tools, with 38% reporting the use of AI tools to learn about a condition or treatment options [23]. However, that study characterized adoption at the population level and did not examine how patients use AI, how they judge its reliability, or how it may shape clinical decision-making. This study complements and extends those findings by showing that engagement is not merely uptake but invokes reflective and structured practice: participants described verification strategies, bounded trust, and selective incorporation of GenAI into advocacy and preparation rather than replacement of clinicians.

Conversely, compared with our convenience sample, the probability sampling of the earlier national survey provides stronger population representativeness. However, by integrating quantitative patterns with qualitative accounts, this study demonstrates that AI use in rare disease contexts may function subtly in patient adoption, rather than merely as simple technological enthusiasm. Together, these studies suggest that rare disease communities may be early adopters and careful interpreters of GenAI.

This study contrasts with the narratives presented in other highly cited studies. For example, recent experimental work using standardized vignettes suggests that access to LLM tools does not automatically improve lay diagnostic or triage accuracy [43]. However, these findings should be treated with caution and should not be interpreted as evidence that patients “believe misinformation.” Rather, such findings may reflect limitations of one-shot vignette paradigms and the difficulty of translating conversational suggestions into a single committed decision, especially when users are not living with symptoms over time. Our study survey adds important nuance: participants reported more cautious and critical GenAI use than is often assumed in clinical and academic commentary. These results underscore the importance of avoiding deficit framings that inadvertently discount patient expertise [42,44].

The study findings should also be read in light of the sample composition. Most respondents were already diagnosed, and many—particularly parents and guardians—were reflecting retrospectively or on a child’s diagnostic pathway, often during a period when GenAI tools were not yet available. The finding that GenAI was not perceived to have shortened the diagnostic journey may, therefore, reflect the timing and structure of the participants’ experiences rather than a limitation of GenAI’s potential to do so—an open question that prospective studies of undiagnosed patients are better placed to address. Future studies designed to capture time since diagnosis directly, and powered for subgroup analysis, could valuably examine whether GenAI use is prompted by diagnoses. Similarly, future work should be powered to explore patient or caregiver age, gender, and education level to explore demographic differences in the adoption of GenAI tools.

The study findings also align with the literature on epistemic injustice in health care [11,12]. Participants used GenAI not to replace clinicians but to translate genetic reports, prepare arguments, and support advocacy during prolonged diagnostic pathways. In this sense, GenAI tools appeared to function as a form of informational support: resources that helped patients to articulate and validate observations that might otherwise be dismissed. However, further qualitative research is needed to substantiate this claim in depth (eg, interviews).

Prior work has emphasized listening to patient concerns as a prerequisite for meaningful clinical recommendations and highlighted how “spetspatients” (expert patients) can reshape health care through knowledge production [9,10]. The study results suggest GenAI may increasingly mediate this process, potentially reducing but also revealing epistemic asymmetries in rare disease encounters. Yet clinicians in rare disease care also operate under genuine, often irreducible, uncertainty [6,45]. Read against this, patient GenAI use can be understood not only as a corrective to epistemic injustice but as a parallel response to a shared epistemic predicament—reframing it as a resource for collaborative management of uncertainty rather than a challenge to clinical authority [46].

It is important to note that the expert role rare disease patients and families take on is often less a chosen empowerment than a burden of necessity, undertaken in the absence of accessible clinical expertise or established evidence. Combined with the epistemic injustice they may encounter if their resulting knowledge is dismissed [12,42,47], rare disease patients and caregivers are placed in a structurally difficult position: they are required to acquire and interpret specialist information, yet may be denied standing as credible knowers when they do so [13,42]. Patient and caregiver use of GenAI should, therefore, be read against this dual pressure, as a tool taken up to manage a burden that the health care system has, in many cases, failed to relieve.

Our findings also align with an established literature documenting the expertise that patients with rare diseases and caregivers develop through both offline networks (such as advocacy organizations and patient registries) and online communities, where peers exchange clinical experiences, navigate uncertainty, and contribute to research agendas [27,48]. Our data extend this picture by showing that GenAI is now being layered onto this existing expertise infrastructure. The conflict this surfaces is not new but is sharpened by GenAI’s reach—patients and caregivers continue to perform substantial epistemic labor that is often unrecognized by health systems, even as the tools available to them become more powerful. This underscores the need for explicit recognition of patient and caregiver expertise in the design and governance of rare disease care, including GenAI’s role within it [20].

Of particular note, the participants in our exploratory survey were cautious about the present use of GenAI, with few reporting harms, yet many believed that these tools could have helped them during earlier stages of rare disease diagnosis. These findings are not contradictory but reflect timing. As noted, GenAI became publicly available only in late 2022, whereas most respondents (93/115, 80.9%) were diagnosed earlier and could not have used it during their diagnostic journey. The retrospective belief that GenAI might have helped therefore reflects unmet need rather than an evaluation of its actual contribution. Given the lengthy diagnostic pathways reported in this study, and for rare disease patients, these attitudes may reflect an adaptive response to unmet clinical need. The nuanced reflections on uptake also challenge dominant narratives that frame patient use of GenAI mainly as a safety concern.

### Strengths and Limitations

This study has several limitations. First, we used a nonprobability convenience sample, which limits generalizability. Participants were recruited through web-based rare disease networks and community organizations, and it is likely that respondents who chose to participate were more digitally literate and more interested in digital tools or GenAI than nonrespondents. Responses were likely biased in favor of highly engaged patients and caregivers who had affiliated with these rare disease networks for information and peer support. Notably, network membership in this context reflects community engagement rather than an advocacy role, and the sample should not be read as composed of designated patient advocates. This is, however, the population in which patterns of early GenAI use are most visible and most relevant for informing emerging patient-facing guidance. Recent national data indicate that rare disease households are disproportionately early adopters of these tools [23], suggesting that our sample represents the leading edge of community uptake rather than an isolated subgroup.

Notwithstanding, and second, our sample was highly educated and based mostly in the United States, and the sample size was modest and not powered to examine associations between respondents’ demographic characteristics and outcomes. In addition, it was not possible to establish thematic saturation due to the limitations of the dataset. Third, to minimize respondent burden and limit the collection of potentially sensitive personal data, the survey included a restricted set of demographic variables and did not collect information on gender, precluding analysis of potential gender-based differences. Fourth, although the qualitative data provided important contextual insights, open-text responses were typically brief, limiting the depth of interpretation and precluding more nuanced qualitative analyses. For example, items such as the use of GenAI for emotional or social support could have been interpreted either as using chatbots for therapy bots, or as seeking out information about services to support patients and caregivers. Furthermore, more targeted interviews or focus groups could disambiguate differing interpretations.

These limitations should be considered alongside important strengths. To our knowledge, this study represents one of the first in-depth patient-centered and family-centered surveys to examine experiences and opinions regarding GenAI use in health care, which focuses specifically on rare disease communities. In addition, when it came to reported rare diseases, our sample was highly heterogeneous. Recruitment was conducted exclusively through validated patient and family networks, rather than open social media platforms, reducing risks of contamination, ineligible participation, and automated or duplicate responses. That said, we acknowledge that 87% of respondents did not state where they learned about the survey; this was an optional question, however, and it may have been perceived as redundant by our participants. The mixed methods design enabled the integration of quantitative patterns with qualitative perspectives, providing a more comprehensive picture of how GenAI tools are currently being used and perceived outside clinical settings.

Taken together, these findings provide early, exploratory evidence of a growing but largely unexamined layer of patient-facing GenAI use. More robust research is needed, including studies using larger and more representative samples, longitudinal designs, and in-depth qualitative methods, such as interviews or focus groups, to better understand patient experiences, potential benefits, risks, and implications for clinical practice and policy. Furthermore, translation of these early findings to wider populations will require complementary work, including plain-language patient guidance, peer-led informational resources distributed through rare disease networks, and clinician communication training, alongside larger and more representative survey samples.

### Conclusions

This study reveals a largely invisible layer of patient-facing GenAI use occurring outside clinical oversight. Patients with rare diseases and their caregivers were not replacing clinicians but were using AI to interpret information, prepare questions, and support advocacy developed through prolonged diagnostic uncertainty.

Although the findings are limited by a modest, highly educated convenience sample and therefore not broadly generalizable, the study provides timely and original insight into how a historically marginalized patient population is engaging with emerging technologies. Rather than overreliance, participants demonstrated cautious and critical use while recognizing AI’s potential to reduce diagnostic delays and informational scarcity. GenAI, therefore, does not merely introduce new risks into rare disease care; it also exposes longstanding epistemic gaps within it and suggests that future governance and clinical practice should be organized around partnership with informed patients rather than re-enacting justifications for paternalistic protectionism.

## Supplementary material

10.2196/93720 Multimedia Appendix 1 Informed consent and survey.

10.2196/93720 Multimedia Appendix 2 Anonymized data.

10.2196/93720 Multimedia Appendix 3 Results by respondent group.

10.2196/93720 Checklist 1 CHERRIES guidelines.

## Abbreviations

CHERRIES: Checklist for Reporting Results of Internet E-Surveys
GenAI: generative artificial intelligence
LLM: large language model
