# Supplementary material for: Patient and Family Perspectives on Generative AI Tools in Rare Diseases: Exploratory Mixed Methods Online Survey
Source: J Particip Med. 2026 Jul 24;18:e93720. doi: 10.2196/93720 (PMC13399968; doi:10.2196/93720)
Supplement: Multimedia Appendix 1 [file jopm-v18-e93720-s001.pdf]

Rare Diseases and Generative AI: Patient & Parent Perspectives Survey

What is the purpose of the study?

This is a research study that seeks to understand how patients with rare diseases, and parents of children with rare diseases, are using or thinking about generative AI tools (such as ChatGPT) in their healthcare journeys. The objective is to explore the experiences, attitudes, and perceived benefits or risks of these technologies in the context of rare disease diagnosis and treatment. The study is intended to inform future research, policy, and practice related to generative AI in healthcare.

How are individuals selected for this research study? How many will participate?

Participants must be:

Adults aged 18 or older
Either diagnosed with a rare disease themselves or the parent/guardian of a child diagnosed with a rare disease
Able to complete an online English-language survey

Participants are identified via public distribution of an anonymous survey link by Combined Brain, a rare disease advocacy organization. The survey will be shared via Combined Brain's email lists, newsletters, and social media to its extensive network of rare disease communities. We aim to collect responses from approximately 100-250 participants, although no strict sample size is enforced due to the voluntary and anonymous nature of the study.

What do I have to do if I'm in the study?

If you participate, you will be asked to complete a brief online survey (approximately 5 minutes) about:

Your diagnostic journey
Any use of generative AI tools (e.g., ChatGPT) in your healthcare experience
Your opinions on the potential benefits or concerns related to generative AI use in rare disease care

Participation is entirely voluntary, and you may skip any question.

What are the risks of this research study?

This is a minimal risk study. Potential risks include:

Mild emotional discomfort when recalling difficult aspects of the diagnostic process

Because the survey is fully anonymous, and no identifiable data (e.g., name, IP address, email) is collected, there no risks to privacy.

What are the benefits of this research study?

There may be no direct benefit to individual participants. However:

You may find personal value in reflecting on your experiences
The insights gathered may benefit others in the rare disease community by guiding future support tools and healthcare policies
The medical and research communities may benefit from increased understanding of how generative AI is perceived and used in rare disease contexts

What will I receive from participating?

There is no compensation or reimbursement for participation. However, the survey is brief (5 minutes), and your input will contribute to valuable research in this underexplored area.

What will happen with the information obtained as part of this research study? What about confidentiality?

All responses will remain strictly anonymous. No personal identifiers (e.g., names, IP addresses, contact information) will be collected. Survey data will be:

Stored securely on password-protected institutional servers
Accessed only by the study team
Used only in aggregate form in presentations or publications

No individual response will be identifiable in any reporting.

If I do not want to take part in this research study, what are the other choices?

Participation is completely voluntary. You may choose not to participate or withdraw at any point before submitting the survey without penalty. Declining to participate will not affect any current or future healthcare services you receive at Boston Children's Hospital or elsewhere.

Who should I contact if I have questions?

If you have questions about the study, you may contact:

Principal Investigator: Kenneth Mandl, MD, MPH
Email: Kenneth.Mandl@childrens.harvard.edu

Please continue to the survey below if you wish to participate.

If you do not wish to participate, you may close this browser window.

Rare Diseases and Generative AI: Patient & Parent Perspectives Survey

Section 1: About You

1. Which best describes you?

◯ I am a patient with a rare disease.
◯ I am a parent/guardian of a child with a rare disease.

---

2. Age group

◯ Under 18
◯ 18-24
◯ 25-34
◯ 35-44
◯ 45-54
◯ 55-64
◯ 65 or older

3. Highest level of education

◯ Primary school
◯ Secondary school
◯ College or vocational training
◯ University degree
◯ Postgraduate degree
◯ Prefer not to say

4. Geographic location (country or region) ______________________

5. Name of rare disease ______________________

Section 2: Diagnostic Journey

6. Approximately how many healthcare professionals did you consult before diagnosis? ______________________

7. Approximately how long did it take from symptom onset to correct diagnosis?

◯ Less than 1 year
◯ 1-2 years
◯ 3-5 years
◯ 6-10 years
◯ More than 10 years

During which year was your rare disease officially diagnosed? ______________________

Section 3: Generative AI Usage

8. Have you used generative AI tools (e.g., ChatGPT, Claude, Perplexity) for managing your/your child's rare disease?

◯ Yes, regularly
◯ Yes, occasionally
◯ Yes, once or twice
◯ No, never

9. For which purposes have you used generative AI? (Select all that apply)

☐ Suggesting possible diagnoses
☐ Finding specialists or care centers
☐ Exploring new treatments or clinical trials
☐ Interpreting medical tests or notes
☐ Preparing questions for healthcare visits
☐ Emotional or social support/advice
☐ Other

10a. Please specify the other purposes for which you have used generative AI ______________________

10. Has generative AI significantly influenced your medical decisions (e.g., prompted new tests or treatments)?

◯ Yes, strongly
◯ Yes, somewhat
◯ No, minimal or no influence

11. Did generative AI directly contribute to your formal medical diagnosis?

◯ Yes, critical contribution
◯ Yes, somewhat helpful
◯ No, did not help
◯ Unsure

12. Did generative AI help shorten your diagnostic journey?

◯ Yes, significantly
◯ Yes, moderately
◯ No, not noticeably
◯ Unsure

13. How trustworthy do you find AI-generated health information compared to traditional sources?

◯ More trustworthy
◯ Equally trustworthy
◯ Less trustworthy
◯ Unsure

14. Have you discussed AI-generated information with your healthcare provider?

◯ Yes, they were supportive
◯ Yes, they were skeptical
◯ No, I have not discussed

Section 4: Concerns and Attitudes

15. How concerned are you about the accuracy of AI-generated medical information?

◯ Not concerned
◯ Slightly concerned
◯ Moderately concerned
◯ Very concerned
◯ Extremely concerned

16. Have you experienced harm or negative outcomes from generative AI advice or information?

◯ Yes
◯ No

16a. Please briefly describe the harm or negative outcomes you experienced from generative AI advice or information ______________________________

17. What factors, if any, have influenced your decision not to use generative AI tools or to use them more cautiously? (Select all that apply)

☐ None. I use generative AI tools without hesitation
☐ Lack of trust
☐ Privacy concerns
☐ Uncertainty about accuracy
☐ Technological comfort/skill
☐ Discouragement by healthcare provider
☐ Lack of awareness or access
☐ Other

17a. Please describe other factors that have influenced your decision not to use generative AI tools or to use them more cautiously. __________________________________

Section 5: Final Reflections

18. Is there anything else you would like to share about your experiences with rare disease care, or your perspectives on the potential role of generative AI in this area? ____

19. Where did you hear about this survey?

◯ Consortium for Outcome Measures and Biomarkers for Neurodevelopmental Disorders (COMBINEDBrain.org)
◯ Global Genes
◯ EveryLife Foundation
◯ Other (please specify)

19a. Please specify where you heard about this survey

____

Thank you for your valuable insights and contribution!

Please click the "Submit" button below to complete the survey.

Internal tracking

Survey duration __________________________________

Submission timestamp __________________________________

Metrics

Survey Duration

____

Submission timestamp

____
