# Supplementary material for: Patient and Family Perspectives on Generative AI Tools in Rare Diseases: Exploratory Mixed Methods Online Survey
Source: J Particip Med. 2026 Jul 24;18:e93720. doi: 10.2196/93720 (PMC13399968; doi:10.2196/93720)
Supplement: Multimedia Appendix 2 [file jopm-v18-e93720-s002.docx]

# Rare Disease and AI Patient Survey Preliminary Results

## Important Links:

URL to full data export via REDCap: <https://redcap.tch.harvard.edu/redcap_edc/redcap_v14.0.43/DataExport/index.php?pid=6000>

Autogenerated descriptive/frequency stats: <https://redcap.tch.harvard.edu/redcap_edc/redcap_v14.0.43/DataExport/index.php?pid=6000&stats_charts=1&report_id=ALL&record=undefined&event_id=&page=rare_diseases_and_generative_ai_patient_parent_per>

Codebook: https://redcap.tch.harvard.edu/redcap_edc/redcap_v14.0.43/Design/data_dictionary_codebook.php?pid=6000

## Summary Tables:

Number of surveys completed: 115

Survey Duration (mean = 5.3 minutes, SD= 3.7 minutes)

Geographical location of respondents: 84.3% from the United States

Average number of healthcare professionals consulted before diagnosis: 7.3, SD= 12.0

| Survey Respondent Characteristics (n=115) | | |
| --- | --- | --- |
|  | Frequency | Percent |
| **Patient/Parent** |  |  |
| I am a parent/guardian of a child with a rare disease. | 74 | 64.3 |
| I am a patient with a rare disease. | 40 | 34.8 |
| Missing | 1 | 0.9 |
|  |  |  |
| **Age group** |  |  |
| 18-24 | 2 | 1.7 |
| 25-34 | 18 | 15.7 |
| 35-44 | 35 | 30.4 |
| 45-54 | 36 | 31.3 |
| 55-64 | 16 | 13.9 |
| 65 or older | 8 | 7 |
|  |  |  |
| **Highest level of education** |  |  |
| College or vocational training | 27 | 23.5 |
| Postgraduate degree | 46 | 40 |
| Secondary school | 1 | 0.9 |
| University degree | 41 | 35.7 |

| Prior use and experience with AI (n=115) | | |
| --- | --- | --- |
|  | **Frequency** | **Percent** |
| **Prior use of generative AI tools** |  |  |
| No, never | 52 | 45.2 |
| Yes, occasionally | 21 | 18.3 |
| Yes, once or twice | 17 | 14.8 |
| Yes, regularly | 25 | 21.7 |
|  |  |  |
| **Purposes generative AI has been used** |  |  |
| Emotional or social support/advice | 18 | 15.7 |
| Preparing questions for healthcare visits | 17 | 14.8 |
| Interpreting medical tests or notes | 37 | 32.2 |
| Exploring new treatments or clinical trials | 53 | 46.1 |
| Finding specialists or care centers | 29 | 25.2 |
| Suggesting possible diagnoses | 28 | 24.3 |
| Other | 37 | 32.2 |
|  |  |  |
| **Influence of AI on medical decisions** |  |  |
| Missing | 1 | 0.9 |
| No, minimal or no influence | 77 | 67 |
| Yes, somewhat | 27 | 23.5 |
| Yes, strongly | 10 | 8.7 |
|  |  |  |
| **Contribution of AI in diagnosis** |  |  |
| Missing | 1 | 0.9 |
| No, did not help | 90 | 78.3 |
| Unsure | 13 | 11.3 |
| Yes, critical contribution | 4 | 3.5 |
| Yes, somewhat helpful | 7 | 6.1 |
|  |  |  |
| **Diagnosis journey shortened due to AI** |  |  |
| Missing | 2 | 1.7 |
| No, not noticeably | 93 | 80.9 |
| Unsure | 9 | 7.8 |
| Yes, moderately | 8 | 7 |
| Yes, significantly | 3 | 2.6 |
|  |  |  |
| **Perceived trustworthiness of AI-generated health information compared to traditional sources** |  |  |
| Equally trustworthy | 33 | 28.7 |
| Less trustworthy | 45 | 39.1 |
| More trustworthy | 7 | 6.1 |
| Unsure | 30 | 26.1 |
|  |  |  |
| **Ever discussed AI-generated information with healthcare provider** |  |  |
| Missing | 2 | 1.7 |
| No, I have not discussed | 90 | 78.3 |
| Yes, they were skeptical | 9 | 7.8 |
| Yes, they were supportive | 14 | 12.2 |
|  |  |  |
| **Concerned with accuracy of AI-generated medical information** |  |  |
| Missing | 1 | 0.9 |
| Extremely concerned | 18 | 15.7 |
| Moderately concerned | 31 | 27 |
| Not concerned | 12 | 10.4 |
| Slightly concerned | 31 | 27 |
| Very concerned | 22 | 19.1 |
|  |  |  |
| **Experience of harm or negative outcomes from generative AI advice or information** |  |  |
| Missing | 3 | 2.6 |
| No | 105 | 91.3 |
| Yes | 7 | 6.1 |
|  |  |  |
| **Factors that have influenced your decision not to use generative AI tools or to use them more cautiously** |  |  |
| None. I use generative AI tools without hesitation | 21 | 18.3 |
| Lack of trust | 35 | 30.4 |
| Privacy concerns | 36 | 31.3 |
| Uncertainty about accuracy | 75 | 65.2 |
| Technological comfort/skill | 11 | 9.6 |
| Discouragement by healthcare provider | 7 | 6.1 |
| Lack of awareness or access | 10 | 8.7 |
| Other | 7 | 6.1 |

## Individual Prompt Response Frequencies:

| **Patient/Parent** | | | | |
| --- | --- | --- | --- | --- |
|  | Frequency | Percent | Valid Percent | Cumulative Percent |
| Missing | 1 | 0.9% | 0.9% | 0.9% |
| I am a parent/guardian of a child with a rare disease. | 74 | 64.3% | 64.3% | 65.2% |
| I am a patient with a rare disease. | 40 | 34.8% | 34.8% | 100% |
| Total | 115 | 100% | 100% |  |

| **Age group** | | | | |
| --- | --- | --- | --- | --- |
|  | Frequency | Percent | Valid Percent | Cumulative Percent |
| 18-24 | 2 | 1.7% | 1.7% | 1.7% |
| 25-34 | 18 | 15.7% | 15.7% | 17.4% |
| 35-44 | 35 | 30.4% | 30.4% | 47.8% |
| 45-54 | 36 | 31.3% | 31.3% | 79.1% |
| 55-64 | 16 | 13.9% | 13.9% | 93% |
| 65 or older | 8 | 7% | 7% | 100% |
| Total | 115 | 100% | 100% |  |

| **Highest level of education** | | | | |
| --- | --- | --- | --- | --- |
|  | Frequency | Percent | Valid Percent | Cumulative Percent |
| College or vocational training | 27 | 23.5 | 23.5 | 23.5 |
| Postgraduate degree | 46 | 40 | 40 | 63.5 |
| Secondary school | 1 | 0.9 | 0.9 | 64.3 |
| University degree | 41 | 35.7 | 35.7 | 100 |
| Total | 115 | 100 | 100 |  |

| **Geographic location country or region** | | | | |
| --- | --- | --- | --- | --- |
|  | Frequency | Percent | Valid Percent | Cumulative Percent |
| Missing | 1 | 0.9% | 0.9% | 0.9% |
| Arizona | 1 | 0.9% | 0.9% | 1.7% |
| Arkansas, USA | 1 | 0.9% | 0.9% | 2.6% |
| Australia | 3 | 2.6% | 2.6% | 5.2% |
| California | 6 | 5.2% | 5.2% | 10.4% |
| Canada | 2 | 1.7% | 1.7% | 12.2% |
| Chicago | 1 | 0.9% | 0.9% | 13.0% |
| Chicagoland | 1 | 0.9% | 0.9% | 13.9% |
| Chicagoland area | 1 | 0.9% | 0.9% | 14.8% |
| Colombia | 1 | 0.9% | 0.9% | 15.7% |
| england | 2 | 1.7% | 1.7% | 17.4% |
| Florida, USA | 1 | 0.9% | 0.9% | 18.3% |
| Hungary | 1 | 0.9% | 0.9% | 19.1% |
| Iowa | 1 | 0.9% | 0.9% | 20.0% |
| Israel | 1 | 0.9% | 0.9% | 20.9% |
| Litauen | 1 | 0.9% | 0.9% | 21.7% |
| Massachusetts | 1 | 0.9% | 0.9% | 22.6% |
| Midwest USA | 3 | 2.6% | 2.6% | 25.2% |
| New England | 1 | 0.9% | 0.9% | 26.1% |
| New York | 1 | 0.9% | 0.9% | 27.0% |
| Northeast USA | 2 | 1.7% | 1.7% | 28.7% |
| PA | 1 | 0.9% | 0.9% | 29.6% |
| pakistan | 1 | 0.9% | 0.9% | 30.4% |
| Republic of California | 1 | 0.9% | 0.9% | 31.3% |
| sc | 1 | 0.9% | 0.9% | 32.2% |
| Southeast | 1 | 0.9% | 0.9% | 33.0% |
| Spain | 1 | 0.9% | 0.9% | 33.9% |
| St. Paul, MN USA | 1 | 0.9% | 0.9% | 34.8% |
| Stamford CT | 2 | 1.7% | 1.7% | 36.5% |
| Texas, USA | 3 | 2.6% | 2.6% | 39.1% |
| turkey | 1 | 0.9% | 0.9% | 40.0% |
| Uk | 1 | 0.9% | 0.9% | 40.9% |
| UK Scotland | 1 | 0.9% | 0.9% | 41.7% |
| United States, Washington State PNW | 1 | 0.9% | 0.9% | 42.6% |
| USA | 66 | 57.4% | 57.4% | 100.0% |
| Total | 115 | 100% | 100% |  |

| **Name of rare disease** | | | | |
| --- | --- | --- | --- | --- |
|  | Frequency | Percent | Valid Percent | Cumulative Percent |
| Missing | 1 | 0.9% | 0.9% | 0.9% |
| Angelman Syndrome | 5 | 4.3% | 4.3% | 5.2% |
| ATP6V1A Encephalopathy | 1 | 0.9% | 0.9% | 6.1% |
| Behçet's disease | 1 | 0.9% | 0.9% | 7.0% |
| Complex Regional Pain Syndrome | 1 | 0.9% | 0.9% | 7.8% |
| Congenital heart disease | 1 | 0.9% | 0.9% | 8.7% |
| Congenital muscular dystrophy- LMNA | 1 | 0.9% | 0.9% | 9.6% |
| DYNC1H1 | 1 | 0.9% | 0.9% | 10.4% |
| dync1h1 related disorder | 1 | 0.9% | 0.9% | 11.3% |
| Ehlers-Danlos Syndrome | 1 | 0.9% | 0.9% | 12.2% |
| Epilesy Sezuires Grandmal | 2 | 1.7% | 1.7% | 13.9% |
| GRIN2B Related Neurodevelopmental Disorder | 1 | 0.9% | 0.9% | 14.8% |
| Hao-Fountain Syndrome | 2 | 1.7% | 1.7% | 16.5% |
| Hunter syndrome | 1 | 0.9% | 0.9% | 17.4% |
| Idiopathic Intracranial Hypertension | 1 | 0.9% | 0.9% | 18.3% |
| IRD's, autism,sphinal miskular diseases, cp etc | 1 | 0.9% | 0.9% | 19.1% |
| IRF2BPL | 1 | 0.9% | 0.9% | 20.0% |
| Joubert syndrome | 1 | 0.9% | 0.9% | 20.9% |
| KCNH1-Related Disorder | 1 | 0.9% | 0.9% | 21.7% |
| kcnq2 | 1 | 0.9% | 0.9% | 22.6% |
| Klippel trenaunay | 1 | 0.9% | 0.9% | 23.5% |
| LAM | 1 | 0.9% | 0.9% | 24.3% |
| Lever's Hereditary Optic Neuropathy (LHON) | 4 | 3.5% | 3.5% | 27.8% |
| LHON | 4 | 3.5% | 3.5% | 31.3% |
| LHON 3460 | 1 | 0.9% | 0.9% | 32.2% |
| Limb Girdle Muscular Dystrophy D1 dnajb6 | 1 | 0.9% | 0.9% | 33.0% |
| LMNA related congenital muscular dystrophy | 1 | 0.9% | 0.9% | 33.9% |
| Lmna-cmd | 1 | 0.9% | 0.9% | 34.8% |
| Loeys Dietz /HCU | 1 | 0.9% | 0.9% | 35.7% |
| Lupus | 1 | 0.9% | 0.9% | 36.5% |
| Malan syndrome | 1 | 0.9% | 0.9% | 37.4% |
| Malignant hyperthermia susceptibility | 1 | 0.9% | 0.9% | 38.3% |
| MED13L Syndrome | 1 | 0.9% | 0.9% | 39.1% |
| MEPAN Syndrome | 1 | 0.9% | 0.9% | 40.0% |
| Mowat-Wilson Syndrome | 1 | 0.9% | 0.9% | 40.9% |
| OCNDS | 1 | 0.9% | 0.9% | 41.7% |
| Ocur chung | 1 | 0.9% | 0.9% | 42.6% |
| OPHN1 Neurodevelopmental Disorder | 1 | 0.9% | 0.9% | 43.5% |
| PCDH19 | 1 | 0.9% | 0.9% | 44.3% |
| Pdcd | 1 | 0.9% | 0.9% | 45.2% |
| PEComa, Tuberous Sclerosis | 1 | 0.9% | 0.9% | 46.1% |
| PWS | 1 | 0.9% | 0.9% | 47.0% |
| ReNU Syndrome | 1 | 0.9% | 0.9% | 47.8% |
| SATB2-Associated Syndrome | 1 | 0.9% | 0.9% | 48.7% |
| SELENON related myopathy | 1 | 0.9% | 0.9% | 49.6% |
| SLC13A5 Epilepsy | 1 | 0.9% | 0.9% | 50.4% |
| SLC6A1 | 2 | 1.7% | 1.7% | 52.2% |
| Spinocerebellar Ataxia Recessive Type 15 | 1 | 0.9% | 0.9% | 53.0% |
| SPS | 1 | 0.9% | 0.9% | 53.9% |
| Stiff leg syndrome | 1 | 0.9% | 0.9% | 54.8% |
| Stiff Person Syndrome | 11 | 9.6% | 9.6% | 64.3% |
| STXBP1 | 1 | 0.9% | 0.9% | 65.2% |
| SYNGAP1 | 5 | 4.3% | 4.3% | 69.6% |
| Syngap1-RD | 1 | 0.9% | 0.9% | 70.4% |
| TRPM3-related neurodevelopmental disorder | 1 | 0.9% | 0.9% | 71.3% |
| TSC | 6 | 5.2% | 5.2% | 76.5% |
| TSC1 | 1 | 0.9% | 0.9% | 77.4% |
| Tuberculosis Sclerosis/Lymphangioleiomyomatosis | 1 | 0.9% | 0.9% | 78.3% |
| tuberous sclerosis complex, RYR1,LGS | 2 | 1.7% | 1.7% | 80.0% |
| Tuberous Sclerosis Complex | 20 | 17.4% | 17.4% | 97.4% |
| warm Autoimmune Hemolytic Anemia | 1 | 0.9% | 0.9% | 98.3% |
| Wong-type dermatomyositis with interstitial lung disease and anti-SRP and -PM/Scl antibodies treated with intravenous immunoglobulin | 1 | 0.9% | 0.9% | 99.1% |
| YWHAG | 1 | 0.9% | 0.9% | 100.0% |
| Total | 115 | 100.0% | 100.0% |  |

| **Approximately how many health care professionals did you consult (mean= 7.3, SD= 12.0)** | | | | |
| --- | --- | --- | --- | --- |
|  | Frequency | Percent | Valid Percent | Cumulative Percent |
| 0 | 1 | 0.9 | 0.9 | 0.9 |
| 1 | 11 | 9.6 | 9.7 | 10.6 |
| 2 | 20 | 17.4 | 17.7 | 28.3 |
| 3 | 14 | 12.2 | 12.4 | 40.7 |
| 4 | 19 | 16.5 | 16.8 | 57.5 |
| 5 | 13 | 11.3 | 11.5 | 69 |
| 6 | 8 | 7 | 7.1 | 76.1 |
| 8 | 2 | 1.7 | 1.8 | 77.9 |
| 9 | 2 | 1.7 | 1.8 | 79.6 |
| 10 | 10 | 8.7 | 8.8 | 88.5 |
| 12 | 2 | 1.7 | 1.8 | 90.3 |
| 15 | 1 | 0.9 | 0.9 | 91.2 |
| 20 | 4 | 3.5 | 3.5 | 94.7 |
| 25 | 1 | 0.9 | 0.9 | 95.6 |
| 30 | 2 | 1.7 | 1.8 | 97.3 |
| 50 | 2 | 1.7 | 1.8 | 99.1 |
| 100 | 1 | 0.9 | 0.9 | 100 |
| Total | 113 | 98.3 | 100 |  |
| Missing | 2 | 1.7 |  |  |
| Total | 115 | 100 |  |  |

| **Approximately how long did it take from symptom onset to correct** | | | | |
| --- | --- | --- | --- | --- |
|  | Frequency | Percent | Valid Percent | Cumulative Percent |
| Missing | 1 | 0.9 | 0.9 | 0.9 |
| 1-2 years | 26 | 22.6 | 22.6 | 23.5 |
| 3-5 years | 15 | 13 | 13 | 36.5 |
| 6-10 years | 12 | 10.4 | 10.4 | 47 |
| Less than 1 year | 45 | 39.1 | 39.1 | 86.1 |
| More than 10 years | 16 | 13.9 | 13.9 | 100 |
| Total | 115 | 100 | 100 |  |

| **During which year was your rare disease officially diagnosed** |  |  |  |  |
| --- | --- | --- | --- | --- |
|  | Frequency | Percent | Valid Percent | Cumulative Percent |
| Missing | 19 | 16.5 | 16.5 | 16.5 |
| 0 (2024) | 1 | 0.9 | 0.9 | 17.4 |
| 1 | 2 | 1.7 | 1.7 | 19.1 |
| 10 | 1 | 0.9 | 0.9 | 20 |
| 13 | 1 | 0.9 | 0.9 | 20.9 |
| 1973 | 1 | 0.9 | 0.9 | 21.7 |
| 1988 | 1 | 0.9 | 0.9 | 22.6 |
| 1993 | 1 | 0.9 | 0.9 | 23.5 |
| 1998 | 3 | 2.6 | 2.6 | 26.1 |
| 2 | 1 | 0.9 | 0.9 | 27 |
| 2 children, 2022, 2024 | 1 | 0.9 | 0.9 | 27.8 |
| 2000 | 1 | 0.9 | 0.9 | 28.7 |
| 2001 | 2 | 1.7 | 1.7 | 30.4 |
| 2002 | 1 | 0.9 | 0.9 | 31.3 |
| 2003 | 1 | 0.9 | 0.9 | 32.2 |
| 2004 | 1 | 0.9 | 0.9 | 33 |
| 2005 | 1 | 0.9 | 0.9 | 33.9 |
| 2009 | 1 | 0.9 | 0.9 | 34.8 |
| 2012 | 2 | 1.7 | 1.7 | 36.5 |
| 2013 | 1 | 0.9 | 0.9 | 37.4 |
| 2014 | 3 | 2.6 | 2.6 | 40 |
| 2015 | 8 | 7 | 7 | 47 |
| 2016 | 2 | 1.7 | 1.7 | 48.7 |
| 2017 | 2 | 1.7 | 1.7 | 50.4 |
| 2018 | 9 | 7.8 | 7.8 | 58.3 |
| 2019 | 3 | 2.6 | 2.6 | 60.9 |
| 2020 | 5 | 4.3 | 4.3 | 65.2 |
| 2021 | 5 | 4.3 | 4.3 | 69.6 |
| 2022 | 4 | 3.5 | 3.5 | 73 |
| 2023 | 6 | 5.2 | 5.2 | 78.3 |
| 2024 | 6 | 5.2 | 5.2 | 83.5 |
| 2025 | 9 | 7.8 | 7.8 | 91.3 |
| 21 | 1 | 0.9 | 0.9 | 92.2 |
| 3 | 1 | 0.9 | 0.9 | 93 |
| 35 | 1 | 0.9 | 0.9 | 93.9 |
| 4 | 2 | 1.7 | 1.7 | 95.7 |
| 4 days old | 1 | 0.9 | 0.9 | 96.5 |
| 7 | 1 | 0.9 | 0.9 | 97.4 |
| Just after first birthday | 1 | 0.9 | 0.9 | 98.3 |
| Suspected in utero and confirmed at birth | 1 | 0.9 | 0.9 | 99.1 |
| teen age years to adult hood | 1 | 0.9 | 0.9 | 100 |
| Total | 115 | 100 | 100 |  |

| **Have you used generative AI tools e.g.ChatGPT, Claude, Perple** | | | | |
| --- | --- | --- | --- | --- |
|  | Frequency | Percent | Valid Percent | Cumulative Percent |
| No, never | 52 | 45.2 | 45.2 | 45.2 |
| Yes, occasionally | 21 | 18.3 | 18.3 | 63.5 |
| Yes, once or twice | 17 | 14.8 | 14.8 | 78.3 |
| Yes, regularly | 25 | 21.7 | 21.7 | 100 |
| Total | 115 | 100 | 100 |  |

| **For which purposes have you used generative AI_Emotional or social support/advice** | | | | |
| --- | --- | --- | --- | --- |
|  | Frequency | Percent | Valid Percent | Cumulative Percent |
| Checked | 18 | 15.7 | 15.7 | 15.7 |
| Unchecked | 97 | 84.3 | 84.3 | 100 |
| Total | 115 | 100 | 100 |  |

| **For which purposes have you used generative AI_Preparing questions for healthcare visits** | | | | |
| --- | --- | --- | --- | --- |
|  | Frequency | Percent | Valid Percent | Cumulative Percent |
| Checked | 17 | 14.8 | 14.8 | 14.8 |
| Unchecked | 98 | 85.2 | 85.2 | 100 |
| Total | 115 | 100 | 100 |  |
|  |  |  |  |  |
|  |  |  |  |  |
| **For which purposes have you used generative AI_Interpreting medical tests or notes** | | | | |
|  | Frequency | Percent | Valid Percent | Cumulative Percent |
| Checked | 37 | 32.2 | 32.2 | 32.2 |
| Unchecked | 78 | 67.8 | 67.8 | 100 |
| Total | 115 | 100 | 100 |  |
|  |  |  |  |  |
|  |  |  |  |  |
| **For which purposes have you used generative AI_Exploring new treatments or clinical trials** | | | | |
|  | Frequency | Percent | Valid Percent | Cumulative Percent |
| Checked | 53 | 46.1 | 46.1 | 46.1 |
| Unchecked | 62 | 53.9 | 53.9 | 100 |
| Total | 115 | 100 | 100 |  |
|  |  |  |  |  |
|  |  |  |  |  |
| **For which purposes have you used generative AI_Finding specialists or care centers** | | | | |
|  | Frequency | Percent | Valid Percent | Cumulative Percent |
| Checked | 29 | 25.2 | 25.2 | 25.2 |
| Unchecked | 86 | 74.8 | 74.8 | 100 |
| Total | 115 | 100 | 100 |  |
|  |  |  |  |  |
|  |  |  |  |  |
| **For which purposes have you used generative AI_Suggesting possible diagnoses** | | | | |
|  | Frequency | Percent | Valid Percent | Cumulative Percent |
| Checked | 28 | 24.3 | 24.3 | 24.3 |
| Unchecked | 87 | 75.7 | 75.7 | 100 |
| Total | 115 | 100 | 100 |  |
|  |  |  |  |  |
|  |  |  |  |  |
| **For which purposes have you used generative AI_Other** | | | | |
|  | Frequency | Percent | Valid Percent | Cumulative Percent |
| Checked | 37 | 32.2 | 32.2 | 32.2 |
| Unchecked | 78 | 67.8 | 67.8 | 100 |
| Total | 115 | 100 | 100 |  |

| **Please specify the other purposes for which you have used generative AI** | | | | |
| --- | --- | --- | --- | --- |
|  | Frequency | Percent | Valid Percent | Cumulative Percent |
| Missing/NA | 79 | 68.7% | 68.7% | 68.7% |
| Advocacy support | 1 | 0.9% | 0.9% | 69.6% |
| advocate IEP, more therapy, fight insurance | 1 | 0.9% | 0.9% | 70.4% |
| behavioral problem treatment, new study, | 1 | 0.9% | 0.9% | 71.3% |
| Centralizing health records for research on Citizen Health | 1 | 0.9% | 0.9% | 72.2% |
| Checking drug-drug interactions; preparing documents (e.g., a letter of medical necessity) and identifying resources (e.g., state ombudsman) to appeal insurance denials for necessary medications and equipment; quickly finding needed info in my son's electronic health records (via Citizen Health's AI Advocate) | 1 | 0.9% | 0.9% | 73.0% |
| Creativity book writing | 1 | 0.9% | 0.9% | 73.9% |
| DDI and potential Rx for co-morbidities that doctors are not paying attention to. | 1 | 0.9% | 0.9% | 74.8% |
| Diet management and recipes | 1 | 0.9% | 0.9% | 75.7% |
| Drafting letters of medical necessity and advocacy work | 1 | 0.9% | 0.9% | 76.5% |
| Fun | 1 | 0.9% | 0.9% | 77.4% |
| grammarly | 1 | 0.9% | 0.9% | 78.3% |
| I just joined Citizens health. I'm in the process of medical record gathering but have not used it for anything else | 1 | 0.9% | 0.9% | 79.1% |
| IEP/school | 1 | 0.9% | 0.9% | 80.0% |
| Insurance Appeal letters and next steps, Letter of Medical Necessity, Pre-Authorizations | 1 | 0.9% | 0.9% | 80.9% |
| insurance management | 1 | 0.9% | 0.9% | 81.7% |
| navigating government benefits | 1 | 0.9% | 0.9% | 82.6% |
| navigating government benefits, identify ng research | 1 | 0.9% | 0.9% | 83.5% |
| Do not use it | 5 | 4.3% | 4.3% | 87.8% |
| nutrition, diet, supplements, physiological pathways, DNA/RNA/protein modeling, drug repurposing, prime editing | 1 | 0.9% | 0.9% | 88.7% |
| Question on diagnosis or terminology | 1 | 0.9% | 0.9% | 89.6% |
| Rare disease research and advocacy activities. | 1 | 0.9% | 0.9% | 90.4% |
| Reading publications, summarizing researcher presentations, understanding the science, running the | 1 | 0.9% | 0.9% | 91.3% |
| Research into molecular function to answer the question of "what exactly is going on to cause these symptoms in my child?"" | 1 | 0.9% | 0.9% | 92.2% |
| Set reminders for appointments and medications | 1 | 0.9% | 0.9% | 93.0% |
| summarising research, checking i understand something, answering medical questions and questions about medications, alternate approaches, writing information for websites, teachers | 1 | 0.9% | 0.9% | 93.9% |
| Treatments, mechanism of action | 1 | 0.9% | 0.9% | 94.8% |
| Understanding drug development, ICD-10 codes, clinical trial design, drafting advocacy letters | 1 | 0.9% | 0.9% | 95.7% |
| Understanding how to translate to medical orovider | 1 | 0.9% | 0.9% | 96.5% |
| Uploading videos of our child to see if they were having epileptic spasms | 1 | 0.9% | 0.9% | 97.4% |
| we create visual aids that are customized to our child's needs .we use skylight to purchase meal plans and manage medical schedules for theripist and core health care | 1 | 0.9% | 0.9% | 98.3% |
| Website development and nonprofit paperwork | 1 | 0.9% | 0.9% | 99.1% |
| work related uses | 1 | 0.9% | 0.9% | 100.0% |
| Total | 115 | 1 | 1 |  |

| **Has generative AI significantly influenced your medical decisions (e.g., prompted new tests or treatments)?** | | | | |
| --- | --- | --- | --- | --- |
|  | Frequency | Percent | Valid Percent | Cumulative Percent |
| Missing | 1 | 0.9 | 0.9 | 0.9 |
| No, minimal or no influence | 77 | 67 | 67 | 67.8 |
| Yes, somewhat | 27 | 23.5 | 23.5 | 91.3 |
| Yes, strongly | 10 | 8.7 | 8.7 | 100 |
| Total | 115 | 100 | 100 |  |
|  |  |  |  |  |
|  |  |  |  |  |
| **Did generative AI directly contribute to your formal medical diagnosis?** | | | | |
|  | Frequency | Percent | Valid Percent | Cumulative Percent |
| Missing | 1 | 0.9 | 0.9 | 0.9 |
| No, did not help | 90 | 78.3 | 78.3 | 79.1 |
| Unsure | 13 | 11.3 | 11.3 | 90.4 |
| Yes, critical contribution | 4 | 3.5 | 3.5 | 93.9 |
| Yes, somewhat helpful | 7 | 6.1 | 6.1 | 100 |
| Total | 115 | 100 | 100 |  |
|  |  |  |  |  |
|  |  |  |  |  |
| **Did generative AI help shorten your diagnostic journey?** | | | | |
|  | Frequency | Percent | Valid Percent | Cumulative Percent |
| Missing | 2 | 1.7 | 1.7 | 1.7 |
| No, not noticeably | 93 | 80.9 | 80.9 | 82.6 |
| Unsure | 9 | 7.8 | 7.8 | 90.4 |
| Yes, moderately | 8 | 7 | 7 | 97.4 |
| Yes, significantly | 3 | 2.6 | 2.6 | 100 |
| Total | 115 | 100 | 100 |  |
|  |  |  |  |  |
|  |  |  |  |  |
| **How trustworthy do you find AI-generated health information compared to traditional sources?** | | | | |
|  | Frequency | Percent | Valid Percent | Cumulative Percent |
| Equally trustworthy | 33 | 28.7 | 28.7 | 28.7 |
| Less trustworthy | 45 | 39.1 | 39.1 | 67.8 |
| More trustworthy | 7 | 6.1 | 6.1 | 73.9 |
| Unsure | 30 | 26.1 | 26.1 | 100 |
| Total | 115 | 100 | 100 |  |
|  |  |  |  |  |
|  |  |  |  |  |
| **Have you discussed AI-generated information with your healthcare provider?** | | | | |
|  | Frequency | Percent | Valid Percent | Cumulative Percent |
| Missing | 2 | 1.7 | 1.7 | 1.7 |
| No, I have not discussed | 90 | 78.3 | 78.3 | 80 |
| Yes, they were skeptical | 9 | 7.8 | 7.8 | 87.8 |
| Yes, they were supportive | 14 | 12.2 | 12.2 | 100 |
| Total | 115 | 100 | 100 |  |
|  |  |  |  |  |
|  |  |  |  |  |
| **How concerned are you about the accuracy of AI-generated medical information?** | | | | |
|  | Frequency | Percent | Valid Percent | Cumulative Percent |
| Missing | 1 | 0.9 | 0.9 | 0.9 |
| Extremely concerned | 18 | 15.7 | 15.7 | 16.5 |
| Moderately concerned | 31 | 27 | 27 | 43.5 |
| Not concerned | 12 | 10.4 | 10.4 | 53.9 |
| Slightly concerned | 31 | 27 | 27 | 80.9 |
| Very concerned | 22 | 19.1 | 19.1 | 100 |
| Total | 115 | 100 | 100 |  |
|  |  |  |  |  |
|  |  |  |  |  |
| **Have you experienced harm or negative outcomes from generative AI advice or information?** | | | | |
|  | Frequency | Percent | Valid Percent | Cumulative Percent |
| Missing | 3 | 2.6 | 2.6 | 2.6 |
| No | 105 | 91.3 | 91.3 | 93.9 |
| Yes | 7 | 6.1 | 6.1 | 100 |
| Total | 115 | 100 | 100 |  |

| **Please briefly describe the harm or negative outcomes you experienced from generative AI advice or information** | | | | |
| --- | --- | --- | --- | --- |
|  | Frequency | Percent | Valid Percent | Cumulative Percent |
| N/A | 109 | 94.8 | 94.8 | 94.8 |
| A person involved in our advocacy group was using it without my knowledge or consent and produced false supposed quotes. Also, it has generated nonexistent references mixing authors and titles and journals, etc. | 1 | 0.9 | 0.9 | 95.7 |
| Did not take into account certain emotions and outliers that change the treatment. ITs not smart enough yet to understand the emotional human side of treatment. | 1 | 0.9 | 0.9 | 96.5 |
| Inaccurate information regarding the drug Idebenone, now used widely, but still found not to be effective, but AI raved about it as though it was a cure. | 1 | 0.9 | 0.9 | 97.4 |
| It has given incorrect information for example the number of exons in the MED13L gene or it has read a genetic report that was a frameshift and deemed it a nonsense. You have to load the AI will the information you want it to pull from ie publications not the internet. It has to be used correctly to be effective | 1 | 0.9 | 0.9 | 98.3 |
| Provided incorrect information about therapy option | 1 | 0.9 | 0.9 | 99.1 |
| w a rare disease the info is not always correct even drs will be misinformed and spread untrue ideas | 1 | 0.9 | 0.9 | 100 |
| Total | 115 | 100 | 100 |  |
|  |  |  |  |  |
|  |  |  |  |  |
| **What factors, if any, have influenced your decision not to use generative AI tools or to use them more cautiously?_None. I use generative AI tools without hesitation** | | | | |
|  | Frequency | Percent | Valid Percent | Cumulative Percent |
| Checked | 21 | 18.3 | 18.3 | 18.3 |
| Unchecked | 94 | 81.7 | 81.7 | 100 |
| Total | 115 | 100 | 100 |  |
|  |  |  |  |  |
|  |  |  |  |  |
| **What factors, if any, have influenced your decision not to use generative AI tools or to use them more cautiously?_Lack of trust** | | | | |
|  | Frequency | Percent | Valid Percent | Cumulative Percent |
| Checked | 35 | 30.4 | 30.4 | 30.4 |
| Unchecked | 80 | 69.6 | 69.6 | 100 |
| Total | 115 | 100 | 100 |  |
|  |  |  |  |  |
|  |  |  |  |  |
| **What factors, if any, have influenced your decision not to use generative AI tools or to use them more cautiously?_Privacy concerns** | | | | |
|  | Frequency | Percent | Valid Percent | Cumulative Percent |
| Checked | 36 | 31.3 | 31.3 | 31.3 |
| Unchecked | 79 | 68.7 | 68.7 | 100 |
| Total | 115 | 100 | 100 |  |
|  |  |  |  |  |
|  |  |  |  |  |
| **What factors, if any, have influenced your decision not to use generative AI tools or to use them more cautiously? _Uncertainty about accuracy** | | | | |
|  | Frequency | Percent | Valid Percent | Cumulative Percent |
| Checked | 75 | 65.2 | 65.2 | 65.2 |
| Unchecked | 40 | 34.8 | 34.8 | 100 |
| Total | 115 | 100 | 100 |  |
|  |  |  |  |  |
|  |  |  |  |  |
| **What factors, if any, have influenced your decision not to use generative AI tools or to use them more cautiously?_Technological comfort/skill** | | | | |
|  | Frequency | Percent | Valid Percent | Cumulative Percent |
| Checked | 11 | 9.6 | 9.6 | 9.6 |
| Unchecked | 104 | 90.4 | 90.4 | 100 |
| Total | 115 | 100 | 100 |  |
|  |  |  |  |  |
|  |  |  |  |  |
| **What factors, if any, have influenced your decision not to use generative AI tools or to use them more cautiously?_ Discouragement by healthcare provider** | | | | |
|  | Frequency | Percent | Valid Percent | Cumulative Percent |
| Checked | 7 | 6.1 | 6.1 | 6.1 |
| Unchecked | 108 | 93.9 | 93.9 | 100 |
| Total | 115 | 100 | 100 |  |
|  |  |  |  |  |
|  |  |  |  |  |
| **What factors, if any, have influenced your decision not to use generative AI tools or to use them more cautiously?_Lack of awareness or access** | | | | |
|  | Frequency | Percent | Valid Percent | Cumulative Percent |
| Checked | 10 | 8.7 | 8.7 | 8.7 |
| Unchecked | 105 | 91.3 | 91.3 | 100 |
| Total | 115 | 100 | 100 |  |
|  |  |  |  |  |
|  |  |  |  |  |
| **What factors, if any, have influenced your decision not to use generative AI tools or to use them more cautiously?_Other** | | | | |
|  | Frequency | Percent | Valid Percent | Cumulative Percent |
| Checked | 7 | 6.1 | 6.1 | 6.1 |
| Unchecked | 108 | 93.9 | 93.9 | 100 |
| Total | 115 | 100 | 100 |  |
|  |  |  |  |  |
|  |  |  |  |  |
| **Please describe other factors that have influenced your decision not to use generative AI tools or to use them more cautiously.** | | | | |
|  | Frequency | Percent | Valid Percent | Cumulative Percent |
| N/A | 109 | 94.8 | 94.8 | 94.8 |
| Have not used | 1 | 0.9 | 0.9 | 95.7 |
| Just having to learn to check its resources but its pretty instant we like Claudia and perplexity chat gpt has to many errors | 1 | 0.9 | 0.9 | 96.5 |
| the environmental impact of the data centers. Making billionaires even richer. | 1 | 0.9 | 0.9 | 97.4 |
| The information is not only extremely unreliable, but AI is very harmful to the environment, while not offering any accurate information | 1 | 0.9 | 0.9 | 98.3 |
| They are destroying the environment and contributing to environmental racism | 1 | 0.9 | 0.9 | 99.1 |
| When using it to research ultra-rare diseases and specifically genetic variants, I have come across several inaccuracies | 1 | 0.9 | 0.9 | 100 |
| Total | 115 | 100 | 100 |  |

| **Is there anything else you would like to share about your experiences with rare disease care, or your perspectives on the potential role of generative AI in this area?** | | | | |
| --- | --- | --- | --- | --- |
|  | Frequency | Percent | Valid Percent | Cumulative Percent |
| Missing | 61 | 53 | 53 | 53 |
| AI helps to find extra information really fast. But I always trust my son doctor first | 1 | 0.9 | 0.9 | 53.9 |
| Again I would emphasize the need to build your own "bot" full of validated data or publications. Don't just allow it to comb the internet freely. You also have to proofread for errors | 1 | 0.9 | 0.9 | 54.8 |
| AI can be use for bad and profit. Companies want to maximize profits and can use data to harm people over profits. MAchines currently are only as good as the programmer who taught them. A misstep or error gets perpetuated like dna over and over. | 1 | 0.9 | 0.9 | 55.7 |
| AI seems to be giving me the information that is popular, but not accurate. | 1 | 0.9 | 0.9 | 56.5 |
| AI should not be used | 1 | 0.9 | 0.9 | 57.4 |
| AI tools are just that, tools. They can help with tedious tasks and reduce the time it takes to search for answers on the internet. For any scientific or technical information I always review the AI sources to make sure they are credible before trusting the data. I've had to correct Gemini so many times when asking about rare disease that I don't trust it for more than overviews. | 1 | 0.9 | 0.9 | 58.3 |
| AI was not around when we were seeking a diagnosis for my son. I firmly believe that if we'd had resources like ChatGPT, I would've been able to push harder against the doctors who were dismissing his symptoms and could've gotten a diagnosis sooner. If anything, AI tools have empowered me to ask questions and make connections that I would otherwise not feel competent to make. I will never use it as an alternative to a medical professional/expert, but it is INCREDIBLY helpful in finding out what may be significant and what may not be. And as a side note, although it was not around when we were attempting to get a diagnosis for my son's TSC, ChatGPT DID tell me I likely had lupus prior to my lupus test coming back positive and receiving the official diagnosis (because I was able to tell it absolutely everything I had going on, whether I thought it was relevant or not). I would never say "I must have lupus" because ChatGPT suggested it as a possibility, but I was able to advocate for autoimmune testing (my doctor didn't think there was anything like that going on) and that ultimately got the diagnosis I needed. | 1 | 0.9 | 0.9 | 59.1 |
| AI wasn't around when my son was diagnosed and now the information is created by my non-profit. It will become more useful over time. | 1 | 0.9 | 0.9 | 60 |
| As generative AI is more and more depended on for medical answers, I worry that healthcare professionals will lose their personal knowledge and become too dependent on a system that could go down or become infected with misinformation through some malicious attempt to corrupt the data by hackers. | 1 | 0.9 | 0.9 | 60.9 |
| At the time of my daughter's diagnosis, this was not available. But I am also a rare disease patient, different one, and do see the value when looking for quick information. For my daughter's condition, helps me refresh what is out there to better discuss with physicians and her teachers. | 1 | 0.9 | 0.9 | 61.7 |
| Every day we pray for a cure and be normal again. | 1 | 0.9 | 0.9 | 62.6 |
| G AI , Needs to be more envolved with patients feeling and more empathy when giving feed back and be more postive | 1 | 0.9 | 0.9 | 63.5 |
| Generative AI has a great potential to democratize scientific knowledge to parents and caregivers (NotebookLM for example does a great job of breaking down dense research papers). It is also going to be part of all drug discovery going forward hopefully finding cures for rare diseases faster than ever before. | 1 | 0.9 | 0.9 | 64.3 |
| Generative AI has taught me about the gene, the related pathway, other genes/disorders associated with the pathway and has helped me identify a gap in care around cognition that I am considering filling via the establishment of a new nonprofit. I feel that generative AI has given me tools and resources to be able to dive deeply into my child's ultra-rare disease whereas traditional MDs/PhDs simply don't have the time to help me understand and answer all of my questions. | 1 | 0.9 | 0.9 | 65.2 |
| I always ask for links or references to verify what it is telling me is accurate | 1 | 0.9 | 0.9 | 66.1 |
| I am hopeful that AI will evolve significantly to address and cure significant rare diseases. I do think it has tremendous potential. Every parent or carrier is desperate for information and more desperate for advancements in medicine in this area. | 1 | 0.9 | 0.9 | 67 |
| I am very interested in the possibilities offered by AI in shortening the diagnosis timeline for CRPS for others. Many CRPS patients have taken 7-10 years for an accurate diagnosis. | 1 | 0.9 | 0.9 | 67.8 |
| I entered all my past medical labs and notes from docs. chatgpt was able to pinpoint my disease and was correct when I finally found a specialist that knew my disease. | 1 | 0.9 | 0.9 | 68.7 |
| I feel the human interaction is the best way to receive care. Human touch is key to connection. It's genuine, not artificial. Artificial would only be for informational use to be discussed by a real personable doctor that you can relate to. | 1 | 0.9 | 0.9 | 69.6 |
| I find that despite advances in AI technology, it is also the lack of providers that know anything about my condition. Trying to locate a specialist has required me to travel out of state for care. I do not believe AI will be able to solve this problem. | 1 | 0.9 | 0.9 | 70.4 |
| I have been very slow to using AI as I will admit that I don't entirely trust the outcomes to not be biased. | 1 | 0.9 | 0.9 | 71.3 |
| I need more help | 1 | 0.9 | 0.9 | 72.2 |
| I really like that I can put in a link to research publications and have them summarized in a way that a person with a non-science background can more easily understand. | 1 | 0.9 | 0.9 | 73 |
| I think generative AI may be better at summarizing information that user pulls from reliable source instead of asking it questions without specifying which sources you'd like it to pull from | 1 | 0.9 | 0.9 | 73.9 |
| I think it has the potential to help counteract human error and physician biases, but also the risk of providing misinformation | 1 | 0.9 | 0.9 | 74.8 |
| I think parents should be very cautious in using AI, especially for emotional support. Having a medically complex child can be very lonely, and leaning too much on AI for support could damage already strained relationships. | 1 | 0.9 | 0.9 | 75.7 |
| I use AI almost every day and in virtually every facet of my life, including for rare disease care management, research, and advocacy activities. I am perfectly happy putting personal information in AI tools because I believe the benefits far outweigh the risks. Obviously, we need to treat AI outputs with a grain of salt, but the same is true for any information that I find on the internet or that I get from my care team. In fact, AI has been more accurate than several of my care providers on a few key issues, but it's far from perfect. | 1 | 0.9 | 0.9 | 76.5 |
| I use generative AI for my professional career all the time, but really have not used it for my son's diagnosis or care up to this point. | 1 | 0.9 | 0.9 | 77.4 |
| I will never trust generative AI as a source when so many wonderful research summaries are available that are written by human scientists. | 1 | 0.9 | 0.9 | 78.3 |
| I will not use generative AI. I believe it does more harm than good. I have experienced many difficulties with my healthcare, but still would not resort to AI for help. | 1 | 0.9 | 0.9 | 79.1 |
| I worked in biomedical informatics for several years. I do not like nor trust AI. | 1 | 0.9 | 0.9 | 80 |
| I would love to read this paper when it is published. I really want to use it tonight to interpret information about pathology, genetics and more but I have concerns about privacy. I want to know what others use it for. today i used it to guide me on how to get more alerts about syngap1 so i dont miss information online. | 1 | 0.9 | 0.9 | 80.9 |
| I've noticed that AI isn't even able to correctly answer simple questions with no stakes and easily found answers. I would not trust my child's healthcare to AI and would not trust a provider who used AI as anything more than a source of an idea to consider either. I would not want AI used to narrow choices in a diagnostic way or to suggest treatment. I am very leery of AI use. | 1 | 0.9 | 0.9 | 81.7 |
| Important to include sources for AI responses and then check those sources. I also use two or more AI tools as cross reference and frequently ask for clarifications or restatements | 1 | 0.9 | 0.9 | 82.6 |
| In order to get good, reliable answers you have to be able to formulate the right questions and criteria for responses. Ask for sources and cross reference and challenge. Not unlike dealing with live people but maybe a little faster. | 1 | 0.9 | 0.9 | 83.5 |
| It's been helpful for asking a million questions the doctors don't have time for, | 1 | 0.9 | 0.9 | 84.3 |
| Its been a bit frustrating when AI ( and the links associated with the AI results) differ in opinion from the care team and the care team is dismissive. | 1 | 0.9 | 0.9 | 85.2 |
| My experiences with rare disease care is very good and my perspectives on the potential role of generative Al in this area are very good and I would use it more in the future. | 1 | 0.9 | 0.9 | 86.1 |
| My experiences with rare disease care is very good in perspectives on the potential role of generative Al in this area. | 1 | 0.9 | 0.9 | 87 |
| My fear is that we will lose control | 1 | 0.9 | 0.9 | 87.8 |
| My son was diagnosed before AI in 2018. But I would have used AI if it were available back then to try and find answers | 1 | 0.9 | 0.9 | 88.7 |
| NA | 1 | 0.9 | 0.9 | 89.6 |
| No | 2 | 1.7 | 1.7 | 91.3 |
| Nooe | 1 | 0.9 | 0.9 | 92.2 |
| Parents need support as caregivers and also with political advocacy. Can AI help with both? | 1 | 0.9 | 0.9 | 93 |
| Still unclear | 1 | 0.9 | 0.9 | 93.9 |
| Talking to Jet GPT is very helpful. You can ask it to explain things and it doesn't have time constraints. I have used this to explore drug-drug interactions because I'm getting drugs from three different doctors. I have used this to look for alternatives to first-generation therapies that doctors are using without thinking through the side effects. And I have used this to suggest supplements. Certainly JET GPT does not replace a doctor, but it is a valuable tool for parents who are often isolated and stuck dealing with a very sick rare disease child that no one doctor is really taking ownership of. | 1 | 0.9 | 0.9 | 94.8 |
| The data on rare diseases vary so that generative AI would not make much of a difference. | 1 | 0.9 | 0.9 | 95.7 |
| the lack of knowledge amongst health care providers and conflicting standards is huge | 1 | 0.9 | 0.9 | 96.5 |
| There's still a lot of work to do. There was a patient who uploaded their exome into ChatGPT and came to our group claiming to have been diagnosed with the disease. We directed them to the correct confirmatory enzyme testing and they left the group upon receiving the enzyme testing results, presumably because the diagnosis was incorrect. | 1 | 0.9 | 0.9 | 97.4 |
| Unsure how to navigate all the AI options as well. | 1 | 0.9 | 0.9 | 98.3 |
| Very helpful in suggesting relevant publications; helpful in searching through medical records | 1 | 0.9 | 0.9 | 99.1 |
| we need ai to help families manage care fight wrongfully insurance denials and sight sources to resources its great to ask questions i cant wait to be able to test with our medical image results as well to help me understand what the neurologist sees when comparing the charting to the image it can be a great tool. | 1 | 0.9 | 0.9 | 100 |
| Total | 115 | 100 | 100 |  |

| **Where did you hear about this survey** | | | | |
| --- | --- | --- | --- | --- |
|  | Frequency | Percent | Valid Percent | Cumulative Percent |
| Missing | 100 | 87 | 87 | 87 |
| Consortium for Outcome Measures and Biomarkers for Neurodevelopmental Disorders (COMBINEDBrain.org) | 1 | 0.9 | 0.9 | 87.8 |
| EveryLife Foundation | 12 | 10.4 | 10.4 | 98.3 |
| Other (please specify) | 2 | 1.7 | 1.7 | 100 |
| Total | 115 | 100 | 100 |  |

| **Survey Duration (mean = 5.3 minutes, SD= 3.7 minutes)** | | | | |
| --- | --- | --- | --- | --- |
| Minutes | Frequency | Percent | Valid Percent | Cumulative Percent |
| 1.62 | 1 | 0.9 | 0.9 |  |
| 1.73 | 2 | 1.7 | 1.9 | 2.8 |
| 1.75 | 1 | 0.9 | 0.9 | 3.7 |
| 1.87 | 1 | 0.9 | 0.9 | 4.6 |
| 1.93 | 1 | 0.9 | 0.9 | 5.6 |
| 2.07 | 1 | 0.9 | 0.9 | 6.5 |
| 2.1 | 1 | 0.9 | 0.9 | 7.4 |
| 2.12 | 2 | 1.7 | 1.9 | 9.3 |
| 2.18 | 1 | 0.9 | 0.9 | 10.2 |
| 2.32 | 1 | 0.9 | 0.9 | 11.1 |
| 2.42 | 1 | 0.9 | 0.9 | 12 |
| 2.45 | 1 | 0.9 | 0.9 | 13 |
| 2.5 | 1 | 0.9 | 0.9 | 13.9 |
| 2.55 | 1 | 0.9 | 0.9 | 14.8 |
| 2.57 | 1 | 0.9 | 0.9 | 15.7 |
| 2.65 | 1 | 0.9 | 0.9 | 16.7 |
| 2.72 | 1 | 0.9 | 0.9 | 17.6 |
| 2.77 | 1 | 0.9 | 0.9 | 18.5 |
| 2.78 | 1 | 0.9 | 0.9 | 19.4 |
| 2.83 | 2 | 1.7 | 1.9 | 21.3 |
| 2.87 | 1 | 0.9 | 0.9 | 22.2 |
| 2.92 | 1 | 0.9 | 0.9 | 23.1 |
| 2.97 | 2 | 1.7 | 1.9 | 25 |
| 2.98 | 2 | 1.7 | 1.9 | 26.9 |
| 3.03 | 1 | 0.9 | 0.9 | 27.8 |
| 3.08 | 1 | 0.9 | 0.9 | 28.7 |
| 3.27 | 1 | 0.9 | 0.9 | 29.6 |
| 3.28 | 1 | 0.9 | 0.9 | 30.6 |
| 3.35 | 1 | 0.9 | 0.9 | 31.5 |
| 3.37 | 1 | 0.9 | 0.9 | 32.4 |
| 3.43 | 1 | 0.9 | 0.9 | 33.3 |
| 3.45 | 2 | 1.7 | 1.9 | 35.2 |
| 3.48 | 1 | 0.9 | 0.9 | 36.1 |
| 3.53 | 1 | 0.9 | 0.9 | 37 |
| 3.55 | 1 | 0.9 | 0.9 | 38 |
| 3.68 | 1 | 0.9 | 0.9 | 38.9 |
| 3.7 | 1 | 0.9 | 0.9 | 39.8 |
| 3.73 | 1 | 0.9 | 0.9 | 40.7 |
| 3.85 | 1 | 0.9 | 0.9 | 41.7 |
| 3.9 | 1 | 0.9 | 0.9 | 42.6 |
| 4.03 | 1 | 0.9 | 0.9 | 43.5 |
| 4.05 | 1 | 0.9 | 0.9 | 44.4 |
| 4.13 | 2 | 1.7 | 1.9 | 46.3 |
| 4.15 | 1 | 0.9 | 0.9 | 47.2 |
| 4.17 | 1 | 0.9 | 0.9 | 48.1 |
| 4.18 | 1 | 0.9 | 0.9 | 49.1 |
| 4.3 | 1 | 0.9 | 0.9 | 50 |
| 4.33 | 2 | 1.7 | 1.9 | 51.9 |
| 4.4 | 1 | 0.9 | 0.9 | 52.8 |
| 4.45 | 1 | 0.9 | 0.9 | 53.7 |
| 4.5 | 1 | 0.9 | 0.9 | 54.6 |
| 4.52 | 1 | 0.9 | 0.9 | 55.6 |
| 4.55 | 1 | 0.9 | 0.9 | 56.5 |
| 4.57 | 1 | 0.9 | 0.9 | 57.4 |
| 4.6 | 1 | 0.9 | 0.9 | 58.3 |
| 4.65 | 1 | 0.9 | 0.9 | 59.3 |
| 4.7 | 2 | 1.7 | 1.9 | 61.1 |
| 4.78 | 1 | 0.9 | 0.9 | 62 |
| 4.83 | 1 | 0.9 | 0.9 | 63 |
| 4.9 | 1 | 0.9 | 0.9 | 63.9 |
| 4.92 | 1 | 0.9 | 0.9 | 64.8 |
| 5.03 | 1 | 0.9 | 0.9 | 65.7 |
| 5.05 | 1 | 0.9 | 0.9 | 66.7 |
| 5.12 | 1 | 0.9 | 0.9 | 67.6 |
| 5.4 | 1 | 0.9 | 0.9 | 68.5 |
| 5.58 | 1 | 0.9 | 0.9 | 69.4 |
| 5.63 | 1 | 0.9 | 0.9 | 70.4 |
| 5.85 | 2 | 1.7 | 1.9 | 72.2 |
| 5.97 | 1 | 0.9 | 0.9 | 73.1 |
| 6.1 | 1 | 0.9 | 0.9 | 74.1 |
| 6.12 | 1 | 0.9 | 0.9 | 75 |
| 6.47 | 1 | 0.9 | 0.9 | 75.9 |
| 6.62 | 1 | 0.9 | 0.9 | 76.9 |
| 6.63 | 1 | 0.9 | 0.9 | 77.8 |
| 6.78 | 1 | 0.9 | 0.9 | 78.7 |
| 7.17 | 1 | 0.9 | 0.9 | 79.6 |
| 7.23 | 1 | 0.9 | 0.9 | 80.6 |
| 7.6 | 1 | 0.9 | 0.9 | 81.5 |
| 7.63 | 1 | 0.9 | 0.9 | 82.4 |
| 7.73 | 1 | 0.9 | 0.9 | 83.3 |
| 7.83 | 1 | 0.9 | 0.9 | 84.3 |
| 7.88 | 1 | 0.9 | 0.9 | 85.2 |
| 8 | 1 | 0.9 | 0.9 | 86.1 |
| 8.52 | 1 | 0.9 | 0.9 | 87 |
| 8.67 | 1 | 0.9 | 0.9 | 88 |
| 9.02 | 1 | 0.9 | 0.9 | 88.9 |
| 9.25 | 1 | 0.9 | 0.9 | 89.8 |
| 9.3 | 1 | 0.9 | 0.9 | 90.7 |
| 9.35 | 1 | 0.9 | 0.9 | 91.7 |
| 10.32 | 1 | 0.9 | 0.9 | 92.6 |
| 10.37 | 1 | 0.9 | 0.9 | 93.5 |
| 11.47 | 1 | 0.9 | 0.9 | 94.4 |
| 12.55 | 1 | 0.9 | 0.9 | 95.4 |
| 13.57 | 1 | 0.9 | 0.9 | 96.3 |
| 14.72 | 1 | 0.9 | 0.9 | 97.2 |
| 15.12 | 1 | 0.9 | 0.9 | 98.1 |
| 15.43 | 1 | 0.9 | 0.9 | 99.1 |
| 28 | 1 | 0.9 | 0.9 | 100 |
| Total | 108 | 93.9 | 100 |  |
| Missing | System | 7 | 6.1 |  |
| Total | 115 | 100 |  |  |
