# Supplementary material for: Patient and Family Perspectives on Generative AI Tools in Rare Diseases: Exploratory Mixed Methods Online Survey
Source: J Particip Med. 2026 Jul 24;18:e93720. doi: 10.2196/93720 (PMC13399968; doi:10.2196/93720)
Supplement: Multimedia Appendix 3 [file jopm-v18-e93720-s003.docx]

**Appendix 4.** Further analyses

**Table 1.** Respondent characteristics by group.

| Characteristic | Patients  (*n* = 40) | Parents/Guardians  (*n* = 74) | **Total**  (*N* = 115) |
| --- | --- | --- | --- |
| **Age**, *n (%)* |  |  |  |
| 18 – 24 years | 1 (2.5%) | 1 (1.4%) | 2 (1.7%) |
| 25 – 34 years | 7 (17.5%) | 10 (13.5%) | 18 (15.7%) |
| 35 – 44 years | 6 (15%) | 29 (39.2%) | 35 (30.4%) |
| 45 – 54 years | 16 (40%) | 20 (27%) | 36 (31.3%) |
| 55 – 64 years | 7 (17.5%) | 9 (12.2%) | 16 (13.9%) |
| 65 years or older | 3 (7.5%) | 5 (6.8%) | 8 (7%) |
| **Highest attained education**, *n (%)* |  |  |  |
| Secondary school | – | 1 (1.4%) | 1 (.9%) |
| College or vocational training | 17 (42.5%) | 10 (13.5%) | 27 (23.5%) |
| University degree | 11 (27.5%) | 30 (40.5%) | 41 (35.7%) |
| Postgraduate degree | 12 (30%) | 33 (44.6%) | 46 (40%) |
| **Country**, *n (%)* |  |  |  |
| USA | 33 (80.5%) | 65 (87.8%) | 98 (85.2% |
| UK | 1 (2.4%) | 3 (4.1%) | 4 (3.5%) |
| Australia | 1 (2.4%) | 2 (2.7%) | 3 (2.6%) |
| Canada | 2 (4.9%) | – | 2 (1.7%) |
| Colombia | 1 (2.4%) | – | 1 (0.9%) |
| Hungary | – | 1 (1.4%) | 1 (0.9%) |
| Israel | 1 (2.4%) | – | 1 (0.9%) |
| Lithuania | – | 1 (1.4%) | 1 (0.9%) |
| Pakistan | – | – | 1 (0.9%) |
| Spain | 1 (2.4%) | – | 1 (0.9%) |
| Turkey | – | 1 (1.4%) | 1 (0.9%) |
| *Missing* | – | 1 (1.4%) | 1 (0.9%) |

*Note:* One respondent did not indicate whether they were a patient or a guardian, so they were excluded from the respective columns but preserved in the Total column.

**Table 2.** Diagnoses and time to diagnosis by group.

| Characteristic | Patients  (*n* = 40) | Parents/Guardians  (*n* = 74) | **Total**  (*N* = 115) |
| --- | --- | --- | --- |
| **Diagnosis**, *n (%)* |  |  |  |
| Tuberous Sclerosis Complex | 10 (25%) | 21 (28.4%) | 31 (26.1%) |
| Stiff Person Syndrome | 13 (32.5%) | – | 13 (11.3%) |
| Leber Hereditary Optic Neuropathy | 4 (10%) | 5 (6.8%) | 9 (7.8%) |
| SYNGAP1-related Disorder | – | 6 (8.1%) | 6 (5.2%) |
| Angelman Syndrome | – | 5 (6.8%) | 5 (4.3%) |
| Okur-Chung syndrome | – | 2 (2.7%) | 2 (1.7%) |
| DYNC1H1-related Disorder | – | 2 (2.7%) | 2 (1.7%) |
| Hao-Fountain Syndrome | – | 2 (2.7%) | 2 (1.7%) |
| SLC6A1-related disorder | – | 2 (2.7%) | 2 (1.7%) |
| Epilepsy (Grandmal) | 2 (5%) | – | 2 (1.7%) |
| Other single-entry diagnoses ^1^ | 12 (30%) | 28 (37.8%) | 41 (35.7%) |
| *Missing* | – | 1 (1.4%) | 1 (0.9%) |
| **Time to diagnosis**, *n (%)* |  |  |  |
| Less than a year | 12 (30%) | 33 (44.6%) | 45 (39.1%) |
| 1 – 2 years | 5 (12.5%) | 21 (28.4%) | 26 (22.6%) |
| 3 – 5 years | 7 (17.5%) | 7 (9.5%) | 15 (13%) |
| 6 – 10 years | 5 (12.5%) | 7 (9.5%) | 12 (10.4%) |
| More than 10 years | 11 (27.5%) | 5 (6.8%) | 16 (13.9%) |
| *Missing* | – | 1 (1.4%) | 1 (0.9%) |

*Note:* One respondent did not indicate whether they were a patient or a guardian, so they were excluded from the respective columns but preserved in the Total column.

^1^ Single-entry diagnoses: YWHAG; ATP6V1A encephalopathy; OPHN1 NDD; SCAR15; Malan; MED13L; PCDH19; LAM; OCNDS; ReNU; SATB2; TRPM3; SLC13A5 epilepsy; MEPAN; GRIN2B; Hunter; STXBP1; KCNQ2; KCNH1; IRF2BPL; Mowat-Wilson; PDCD; congenital heart disease; Behçet; PWS; Klippel-Trenaunay; Ehlers-Danlos; lupus; warm AIHA; LGMD D1; Wong-type dermatomyositis; CRPS; Loeys-Dietz/HCU; IIH; Joubert; SELENON myopathy; malignant hyperthermia susceptibility; autism.

**Table 3.** Use of GenAI tools by group.

| Characteristic | Patients  (*n* = 40) | Parents/Guardians  (*n* = 74) | **Total**  (*N* = 115) |
| --- | --- | --- | --- |
| **Prior use of GenAI tools**, *n (%)* |  |  |  |
| Never | 20 (50%) | 31 (41.9%) | 52 (45.2%) |
| Occasionally | 5 (12.5%) | 16 (21.6%) | 21 (18.3%) |
| Once or twice | 6 (15%) | 11 (14.9%) | 17 (14.8%) |
| Regularly | 9 (22.5%) | 16 (21.6%) | 25 (21.7%) |

*Note:* One respondent did not indicate whether they were a patient or a guardian, so they were excluded from the respective columns but preserved in the Total column.

**Table 4.** Influence of GenAI tools on medical decisions and diagnosis by group.

| Characteristic | Patients  (*n* = 40) | Parents/Guardians  (*n* = 74) | **Total**  (*N* = 115) |
| --- | --- | --- | --- |
| **Influence on medical decisions**, *n (%)* | |  |  |
| Minimal or no influence | 26 (65%) | 50 (67.6%) | 77 (67%) |
| Somewhat | 7 (17.5%) | 20 (27%) | 27 (23.5%) |
| Strongly | 7 (17.5%) | 3 (4.1%) | 10 (8.7%) |
| *Missing* | – | 1 (1.4%) | 1 (0.9%) |
| **Contribution to formal diagnosis**, *n (%)* | |  |  |
| Did not help | 25 (62.5%) | 65 (87.8%) | 90 (78.3%) |
| Somewhat helpful | 3 (7.5%) | 3 (4.1%) | 7 (6.1%) |
| Critical contribution | 3 (7.5%) | 1 (1.4%) | 4 (3.5%) |
| Unsure | 8 (20%) | 5 (6.8%) | 13 (11.3%) |
| *Missing* | 1 (2.5%) | – | 1 (0.9%) |
| **Shortened diagnostic journey**, *n (%)* | |  |  |
| Not noticeably | 28 (70%) | 65 (87.8%) | 93 (80.9%) |
| Moderately | 4 (10%) | 3 (4.1%) | 8 (7%) |
| Significantly | 2 (5%) | 1 (1.4%) | 3 (2.6%) |
| Unsure | 5 (12.5%) | 4 (5.4%) | 9 (7.8%) |
| *Missing* | 1 (2.5%) | 1 (1.4%) | 2 (1.7%) |

*Note:* One respondent did not indicate whether they were a patient or a guardian, so they were excluded from the respective columns but preserved in the Total column.

**Table 5.** Trust, concerns, and communication regarding GenAI tools by group.

| Characteristic | Patients  (*n* = 40) | Parents/Guardians  (*n* = 74) | **Total**  (*N* = 115) |
| --- | --- | --- | --- |
| **Perceived trustworthiness of AI-generated health information compared to traditional sources**, *n (%)* | | | |
| Less trustworthy | 19 (47.5%) | 25 (33.8%) | 45 (39.1%) |
| Equally trustworthy | 8 (20%) | 25 (33.8%) | 33 (28.7%) |
| More trustworthy | 4 (10%) | 3 (4.1%) | 7 (6.1%) |
| Unsure | 9 (22.5%) | 21 (28.4%) | 30 (26.1%) |
| **Concerns with accuracy of AI-generated medical information**, *n (%)* | | | |
| Extremely concerned | 7 (17.5%) | 11 (14.9%) | 18 (15.7%) |
| Very concerned | 8 (20%) | 13 (17.6%) | 22 (19.1%) |
| Moderately concerned | 10 (25%) | 21 (28.4%) | 31 (27%) |
| Slightly concerned | 8 (20%) | 23 (31.1%) | 31 (27%) |
| Not concerned | 7 (17.5%) | 5 (6.8%) | 12 (10.4%) |
| *Missing* | – | 1 (1.4%) | 1 (0.9%) |
| **Discussions of AI-generated information with healthcare provider**, *n (%)* | | | |
| Not discussed | 29 (72.5%) | 60 (81.1%) | 90 (78.3%) |
| Healthcare provider was sceptical | 5 (12.5%) | 4 (5.4%) | 9 (7.8%) |
| Healthcare provider was supportive | 5 (12.5%) | 9 (12.2%) | 14 (12.2%) |
| *Missing* | 1 (2.5%) | 1 (1.4%) | 1 (0.9%) |

*Note:* One respondent did not indicate whether they were a patient or a guardian, so they were excluded from the respective columns but preserved in the Total column.

**Table 6.** Experience of harm or negative outcomes related to GenAI tools by group.

| Characteristic | Patients  (*n* = 40) | Parents/Guardians  (*n* = 74) | **Total**  (*N* = 115) |
| --- | --- | --- | --- |
| **Experience of harm or negative outcomes from GenAI advice or information**, *n (%)* | | | |
| Yes | 3 (7.5%) | 4 (5.4%) | 7 (6.1%) |
| No | 36 (90%) | 68 (91.9%) | 105 (91.3%) |
| Missing | 1 (2.5%) | 2 (2.7%) | 3 (2.6%) |

*Note:* One respondent did not indicate whether they were a patient or a guardian, so they were excluded from the respective columns but preserved in the Total column.
