# Supplementary material for: Patient and Family Perspectives on Generative AI Tools in Rare Diseases: Exploratory Mixed Methods Online Survey
Source: J Particip Med. 2026 Jul 24;18:e93720. doi: 10.2196/93720 (PMC13399968; doi:10.2196/93720)
Supplement: Checklist 1 [file jopm-v18-e93720-s004.docx]

**Appendix** **2.** Checklist for Reporting Results of Internet E-Surveys (CHERRIES)

| ***Item Category*** | ***Checklist item*** | ***Described in the manuscript*** | ***Cited from the manuscript*** |
| --- | --- | --- | --- |
| **Design** | Describe survey design | Yes | In **Methods** section |
| **IRB (Institutional Review Board) approval and informed consent process** | IRB approval | Yes | **Methods > Study Design and Ethics**  The study received ethics approval from the Boston Children’s Hospital Institutional Review Board (approval ID: # IRB-P00051505) **p.7** |
|  | Informed consent | Yes | **Methods** > **Study Design and Ethics**  Electronic informed consent was obtained from all participants prior to survey initiation.**p.7** |
|  | Data protection | Yes | **Methods** > **Data Collection**  The survey was hosted on REDCap, a secure web-based data capture platform. **p.8** |
| **Development and pre-testing** | Development and testing | Yes | **Methods > Study Development** Survey content was informed by prior literature on patient information-seeking behaviors, emerging scholarship on GenAI in healthcare, and consultation with rare disease community stakeholders [18–20]. In addition, the survey was co-produced with a patient partner with lived experience of rare disease (CEB), who contributed to item development, wording, and assessment of relevance. Draft versions of the survey were reviewed by contacts within participating rare disease networks to assess clarity, accessibility, and face validity, and the instrument was timed to ensure feasibility of completion within approximately five minutes. **p.7** |
| **Recruitment process and description of the sample having access to the questionnaire** | Open survey versus closed survey | Yes | **Methods> Participants and Recruitment**  To intentionally reduce the risks of bot contamination, duplicate participation, and unverifiable eligibility, the survey team and contacts were requested not to share the survey link via any open social media platforms. **p.8** |
|  | Contact mode | Yes | **Methods** **> Participants and Recruitment**  Recruitment used convenience sampling through the established rare disease advocacy networks and community organizations COMBINEBrain [21] and Every Life Foundation [22]. Based in the United States, both are umbrella networks of rare genetic disease communities with validated patient and family member contacts. **p.8** |
|  | Advertising the survey | Yes | **Methods> Participants and Recruitment**  Via email members of these organizations were invited to distribute an anonymous survey link via their email lists and closed network newsletters, and with two requested reminder emails sent during the recruitment period. **p.8** |
| **Survey administration** | Web/E-mail | Yes | **Methods> Participants and Recruitment**  Via email members of these organizations were invited to distribute an anonymous survey link via their email lists and closed network newsletters, and with two requested reminder emails sent during the recruitment period. **p.8** |
|  | Context | Yes | **Methods> Participants and Recruitment**  Via email members of these organizations were invited to distribute an anonymous survey link via their email lists and closed network newsletters, and with two requested reminder emails sent during the recruitment period. To intentionally reduce the risks of bot contamination, duplicate participation, and unverifiable eligibility, the survey team and contacts were requested not to share the survey link via any open social media platforms. **p.8**  **Methods> Data Collection**  The survey was hosted on REDCap, a secure web-based data capture platform. **p.8** |
|  | Mandatory/voluntary | Yes | **Methods>Study Design and Ethics** Participation was voluntary, and respondents could withdraw at any time before submitting the survey. **p.7** |
|  | Incentives | Yes | **Methods>Study Design and Ethics** In addition, there was no compensation for completing the survey. **p.7** |
|  | Time/Date | Yes | **Methods> Data Collection** Data collection occurred between 1 November 2025 and 10 January 2026. **p.8** |
|  | Randomization of items or questionnaires | No |  |
|  | Adaptive questioning | No |  |
|  | Number of Items | Yes | **Methods> Survey Development**  The final survey comprised 19 primary items, including multiple-choice, Likert-type, and checkbox questions, together with four open-text free-response questions. **p.7** |
|  | Number of screens (pages) | No | **N/A** |
|  | Completeness check | No | **N/A** |
|  | Review step | No | **N/A** |
| **Response rates** | Unique site visitor | No | **N/A** |
|  | View rate (Ratio of unique survey visitors/unique site visitors) | No | **N/A** |
|  | Participation rate (Ratio of unique visitors who agreed to participate/unique first survey page visitors) | No | **N/A** |
|  | Completion rate (Ratio of users who finished the survey/users who agreed to participate) | No | **N/A** |
| **Preventing multiple entries from the same individual** | Cookies used | No | **N/A** |
|  | IP check | Yes | **Methods> Data Collection**  No personally identifiable information, including names, email addresses, or IP addresses, was collected, and responses were anonymous. **p.8** |
|  | Log file analysis | No | **N/A** |
|  | Registration | No | **N/A** |
| **Analysis** | Handling of incomplete questionnaires | No | **N/A** |
|  | Questionnaires submitted with an atypical timestamp | No | **N/A** |
|  | Statistical correction | No | **N/A** |

**Reference:**

Eysenbach G. Improving the quality of Web surveys: the Checklist for Reporting Results of Internet E-Surveys (CHERRIES). J Med Internet Res 2004;6:e34.
